# Leaky ribosomal scanning enables tunable translation of bicistronic ORFs in green algae

**DOI:** 10.1073/pnas.2417695122

**Published:** 2025-02-26

**Authors:** Marco A. Dueñas, Rory J. Craig, Sean D. Gallaher, Jeffrey L. Moseley, Sabeeha S. Merchant

**Affiliations:** ^a^Department of Plant and Microbial Biology, University of California, Berkeley, CA 94720; ^b^California Institute for Quantitative Biosciences (QB3), University of California, Berkeley, CA 94720; ^c^Department of Molecular and Cell Biology, University of California, Berkeley, CA 94720; ^d^Division of Environmental Genomics and Systems Biology, Lawrence Berkeley National Laboratory, Berkeley, CA 94720

**Keywords:** Iso-Seq, ribosome profiling, dicistronic, uORFs, transgene expression

## Abstract

Advances in sequencing technology have unveiled examples of nucleus-encoded polycistrons, once considered rare. Exclusively polycistronic transcripts are prevalent in green algae, although the mechanism by which multiple polypeptides are translated from a single transcript is unknown. Here, we used bioinformatic and in vivo mutational analyses to evaluate competing mechanistic models for translation of bicistronic mRNAs in green algae. High-confidence manually curated datasets of bicistronic loci from two divergent green algae, *Chlamydomonas reinhardtii* and *Auxenochlorella protothecoides*, revealed a preference for weak Kozak-like sequences for ORF 1 and an underrepresentation of potential initiation codons before the ORF 2 start codon, which are suitable conditions for leaky ribosome scanning to allow ORF 2 translation. We used mutational analysis in *A. protothecoides* to test the mechanism. In vivo manipulation of the ORF 1 Kozak-like sequence and start codon altered reporter expression at ORF 2, with a weaker Kozak-like sequence enhancing expression and a stronger one diminishing it. A synthetic bicistronic dual reporter demonstrated inversely adjustable activity of green fluorescent protein expressed from ORF 1 and luciferase from ORF 2, depending on the strength of the ORF 1 Kozak-like sequence. Our findings demonstrate that translation of multiple ORFs in green algal bicistronic transcripts is consistent with episodic leaky scanning of ORF 1 to allow translation at ORF 2. This work has implications for the potential functionality of upstream open reading frames (uORFs) found across eukaryotic genomes and for transgene expression in synthetic biology applications.

## Significance

Textbook dogma states that nucleus-encoded genes are monocistronic, producing transcripts with a single translated open reading frame (ORF). However, highly conserved bicistronic loci are pervasive in green algae that are separated by several hundred million years of evolution, speaking to their ancestral origins and functions within the Chlorophyte lineage. A combination of bioinformatic analysis and in vivo gene manipulation supports leaky ribosomal scanning as the primary mechanism for translation of more than one ORF from bicistronic transcripts. We have successfully tuned synthesis levels of two proteins encoded on one mRNA by modifying the ORF 1 Kozak-like sequence and limiting the number of AUG codons upstream of ORF 2. These findings may have broad applications in synthetic biology.

Eukaryotic translation initiation begins at the 5′ cap, where eukaryotic initiation factors (eIFs), the initiator tRNA, and the 40S ribosomal subunit converge to form the 43S preinitiation complex (1). Once assembled, the preinitiation complex scans downstream, seeking a suitable translation initiation site. This is generally discerned by the presence of an initiator codon (AUG) and its surrounding sequence (2). The optimum sequence, referred to as the Kozak consensus, impacts the efficiency of recognition of the initiator codon by the preinitiation complex, and features motifs that are conserved throughout eukaryotes (3–5). After identifying an appropriate initiator codon, 80S assembly, polypeptide elongation, termination, and ribosome disassembly proceed (6). The cap-dependent nature and unidirectional scanning of the preinitiation complex typically prioritizes the first start codon encountered, limiting the recognition of potential downstream ORFs (7, 8). Consequently, eukaryotic nuclear genes have traditionally been considered monocistronic; encoding mature mRNAs with a single protein-coding ORF.

Polycistronic expression refers to the situation in which two (bicistronic) or more ORFs are encoded by a single messenger RNA (mRNA). Although historically considered incompatible with eukaryotic translation, discoveries of nucleus-encoded loci that produce more than one protein have challenged this dogma. Polycistrons in which mature mRNAs contain two or more protein-coding ORFs have been detected in the transcriptomes of fungi (9), plants (10, 11), fruit flies (12, 13), and mammals (14). In many of these instances, the candidate loci are not exclusively polycistronic, with a population of monocistronic transcripts for each ORF also being produced, which has raised questions about the biological importance of polycistronic mRNAs. Moreover, the mechanism by which the downstream ORFs in polycistronic mRNAs are translated remains unknown in most of these cases.

Eukaryotic viruses have evolved unique mechanisms for noncanonical translation of polycistronic mRNAs. An Internal Ribosome Entry Site (IRES) in a mammalian viral RNA can adopt a distinct secondary structure that directly recruits translation initiation components to an ORF, independent of the 5′ cap (15). 2A “self-cleaving” peptide sequences contain motifs that trigger ribosomal skipping events, generating multiple polypeptides from a single ORF due to failure to form a peptide bond (16). Polycistronic expression is an attractive tool for coexpression of transgenes (17), and both IRESs and 2A peptides have been widely used in expression vectors for producing multiple polypeptides (18, 19).

In eukaryotic genomes, variations from canonical translation can drive downstream ORF expression. In cases where two ORFs are in close proximity, ribosome components can reassemble and initiate translation of a downstream ORF following termination at the upstream ORF, a process termed posttermination reinitiation (20–22). In leaky ribosomal scanning, a suboptimal context sequence surrounding the initiator codon allows the scanning complex to occasionally bypass the first ORF, enabling translation of an alternative downstream ORF (22–24). These two competing models for translation of polycistronic ORFs can be distinguished by the role that is played by the sequence surrounding the initiator codon, referred to as the Kozak-like sequence. In the posttermination reinitiation model, a more favorable Kozak-like sequence of the most upstream ORF will increase translation of both ORFs. Conversely, in the leaky ribosome-scanning model, a more favorable Kozak-like sequence of the upstream ORF will increase its translation at the expense of a downstream ORF.

Green algae (Chlorophyta) are a diverse clade of eukaryotes within the plant lineage (25). Like plants, they are primary producers that contribute significantly to carbon capture. Moreover, a few species serve as invaluable reference organisms for understanding metabolic processes such as photosynthesis (26) and are promising platforms for synthetic biology applications (27). We previously used long-read RNA sequencing (i.e. Iso-Seq) to identify widespread and highly conserved loci encoding exclusively polycistronic mature mRNA in diverse green algae (28). Polycistronic genes contain ORFs that are independently translated, containing their own start and stop codons separated by a small spacer sequence (referred to as the “inter-ORF” region). Subsequent sequence analysis and proteomics confirmed the authenticity and translation of multiple ORFs at these loci in the algae, and raised the question of how the downstream ORFs are translated when they occur only in a polycistronic context. Polycistronic ORFs in the green algae vary in length and are often not read in the same frame. These features are incompatible with mechanisms such as 2A peptide motifs or stop codon read-through, and there is no evidence that the inter-ORFs have any IRES-like activity (28). Additionally, in vitro transcription and translation of bicistronic loci from *Chlamydomonas reinhardtii* and *Chromochloris zofingiensis* revealed that the ratio of polypeptides produced from each ORF was dependent on the quality of the upstream (ORF 1) Kozak-like sequence (28). This observation suggests that translation of the downstream ORF (ORF 2) may depend on leaky scanning of the ORF 1 initiator codon, but this was not tested in vivo. In this work, we utilized two divergent (~700 Mya) green algal species, the classic reference organism *C. reinhardtii* and the emerging model alga *Auxenochlorella protothecoides,* to establish a high confidence dataset of bicistronic loci. We then used bioinformatic and in vivo mutational analyses to test the leaky scanning hypothesis as a mechanism for translation of bicistronic ORFs.

## Results

### Establishing a High Confidence Dataset of Polycistronic Loci in Two Divergent Algae.

Previous structural annotation improvements identified 87 loci within the *C. reinhardtii* genome that were exclusively polycistronic (28). We refined this set to identify a high-confidence subset using the following criteria: 1) evidence for translation of ORFs based on ribosome profiling (29), and 2) conservation of the ORFs between the reference strain genome (CC-4532) and two divergent field isolate genomes (CC-1952 and CC-2931) (30). This produced a refined set of 36 high-confidence bicistronic loci (Dataset S1 A and C), 31 of which were previously identified (28). The five additional genes include a deeply conserved bicistronic locus, originally described in land plants, encoding the CDC26 cell cycle regulator and the TTM3 phosphatase (11).

**Fig. 1.**
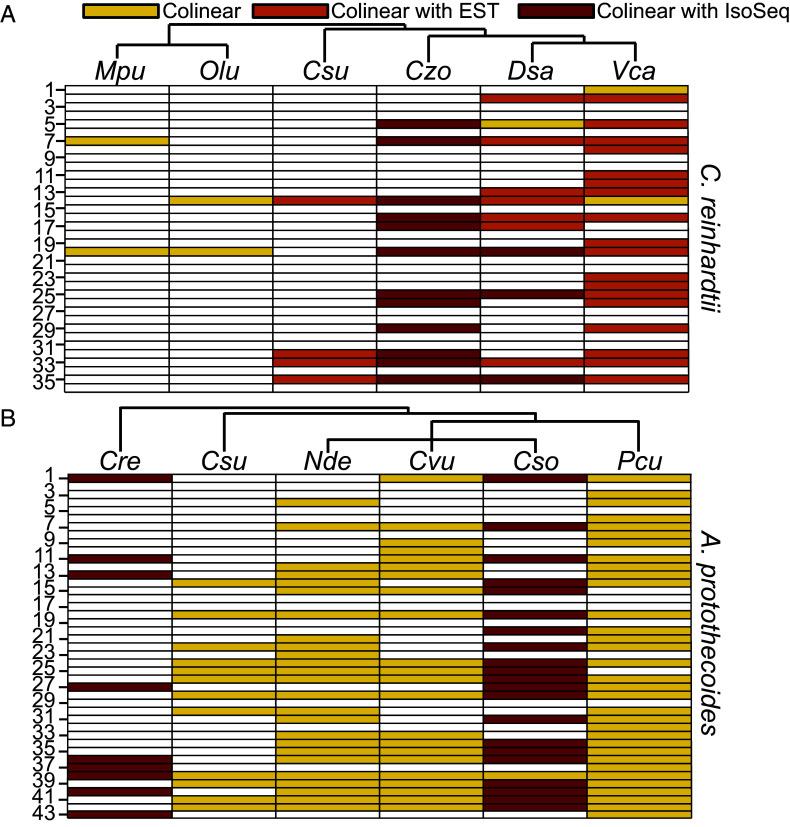
Bicistronic loci in divergent green algae. (*A*) 36 bicistronic loci in *C. reinhardtii* are conserved in field isolates. Translation of both ORFs is supported by Ribo-Seq data. Orthologous bicistronic transcripts were identified in other Chlorophytes. High confidence hits and evidence of colinearity were further evaluated (*Materials and Methods*). Yellow denotes a colinear pair of ORFs with significant similarity to a pair of bicistronic ORFs from *C. reinhardtii*. Red and orange, respectively, indicate additional support with Iso-Seq or EST data. Columns are ordered by the phylogenetic tree above each panel. Species abbreviations (From leftmost to rightmost column) are as follows: *Mpu, Micromonas. pusilla; Olu, Ostreococcus lucimarinus; Csu, Coccomyxa subellipsoidea; Czo, Chromochloris zofingiensis; Dsa, Dunaliella salina; Vca, Volvox carteri.* (*B*) 43 bicistronic loci were identified in *A. protothecoides*. A similar search for orthologs was performed as described in panel *A* but restricted to the Trebouxiophyceae with *C. reinhardtii* as an outgroup. Species abbreviations (from leftmost to rightmost column) are as follows: *Cre, Chlamydomonas reinhardtii; Csu, Coccomyxa subellipsoidea; Nde, Nannochloris desiccata; Cvu, Chlorella vulgaris; Cso, Chlorella sorokiniana; Pcu, Prototheca cutis.*

The predicted amino acid sequences of the ORFs were used to search for bicistronic orthologs in six other chlorophyte species: *Volvox carteri*, *Dunaliella salina* and *Chromochloris zofingiensis* (all Chlorophyceae), *Coccomyxa subellipsoidea* (Trebouxiophyceae), and *Ostreococcus lucimarinus* and *Micromonas pusilla* (Mamiellophyceae). Preservation of the bicistronic arrangement was inferred when pairs of orthologous ORFs were colinear (neighboring ORFs on the same strand) in the queried genome (Fig. 1*A* and Dataset S2A). Out of 36 loci, 21 were bicistronic in at least one of the six chlorophytes. Validation that 20 of these 21 colinear loci were bicistronic was provided by Iso-Seq reads [*C. zofingiensis* (31) and *D. salina* (32)] and/or expressed sequence tags (ESTs) [*V. carteri*, *D. salina*, and *C. subellipsoidea* (28)], confirming conservation of bicistronic organization across hundreds of millions of years of evolution.

For reverse genetic analysis of polycistronic loci, we turned to the unicellular trebouxiophyte alga *A. protothecoides*, which we have developed as a reference organism because of its small nuclear genome and experimental capability for genome manipulation by targeted homologous recombination (33). Using Iso-Seq, we manually searched for loci in *A. protothecoides* where two or more ORFs were exclusively found on the same transcript. This uncovered 43 bicistronic loci within the genome of *A. protothecoides* strain UTEX 250-A (Dataset S1B), most of which had not been previously identified as polycistronic in other species.

The criteria described above were used to assess whether the bicistronic arrangement was conserved in other members of the Trebouxiophyceae: *C. subellipsoidea*, *Chlorella vulgaris*, *Chlorella sorokiniana*, *Nannochloris desiccata*, and *Prototheca cutis*; and in *C. reinhardtii* (Fig. 1*B* and Dataset S2B). 37 out of the 43 bicistronic loci in *A. protothecoides* had colinear hits in at least one other trebouxiophyte, suggesting that they may indeed function as bicistrons. 24 of these loci were further validated by Iso-Seq reads in the transcriptomes of either *C. sorokiniana* (34) or *C. reinhardtii* (28). Notably, nine loci are bicistronic in both *C. reinhardtii* and *A. protothecoides,* indicative of conservation over 700 million years of evolution (35). The 36 bicistronic loci from *C. reinhardtii* and 43 from *A. protothecoides* represent the high confidence set for downstream bioinformatic analyses.

### Structural Features of Green Algal Bicistronic Genes.

We next examined the structural properties of the curated sets of bicistronic loci to identify features differentiating them from monocistronic genes, reasoning that such features might give insight into the mechanism. In *C. reinhardtii*, bicistronic ORF 1 sizes are significantly smaller (median = 357 nt) than ORF 2 sizes (median = 1,113 nt) or monocistronic ORFs (median = 1,509 nt). ORF 1 sequences in the high confidence set are also shorter than previously described in the unrefined set (32) (median = 600 nt) (*SI Appendix*, Fig. S1*B*). This differential was also present in *A. protothecoides*, with ORF 1 (median = 291 nt) significantly shorter than both ORF 2 (median = 1,185 nt) or monocistronic ORFs (median = 1,005 nt) (*SI Appendix*, Fig. S1*E*).

We also compared the distribution of the inter-ORF lengths for colinear genes (defined here as genes on the same chromosome strand with ≤20,000-nt separation between ORFs), and predicted uORFs (small ORFs found in the 5′ untranslated regions of monocistronic genes). Consistent with our previous study (28), the inter-ORF spacing at bicistronic loci is much shorter than the distance between monocistronic colinear genes, both in *C. reinhardtii* (bicistronic median = 183 nt, monocistronic median = 3,415 nt), and *A. protothecoides* (bicistronic median = 40 nt, monocistronic median = 2,518 nt) (*SI Appendix*, Fig. S1 *C* and *F*). The inter-ORF spacing of bicistronic genes from the refined *C. reinhardtii* and *A. protothecoides* sets were not statistically different from uORF-ORF spacing in either genome (median = 148 nt for *C. reinhardtii* and median = 76 nt for *A. protothecoides*) (*SI Appendix*, Fig. S1 *C* and *F*).

Short 5′ UTRs or initiator codons very close to the 5′ cap have been associated with a higher degree of leaky scanning (36, 37). Bicistronic mRNA transcripts have significantly shorter 5′ UTRs (median = 83 nt for *C. reinhardtii*, and median = 54 nt for *A. protothecoides*) than do monocistronic ORFs (median = 214 nt for *C. reinhardtii*, and median = 112 nt for *A. protothecoides*), which may contribute to more frequent leaky ribosomal scanning to bypass the ORF 1 initiation site for downstream ORF translation (*SI Appendix*, Fig. S1 *A* and *D*).

### Bicistronic Transcripts have Weak Kozak-Like Sequence Associated with ORF 1.

**Fig. 2.**
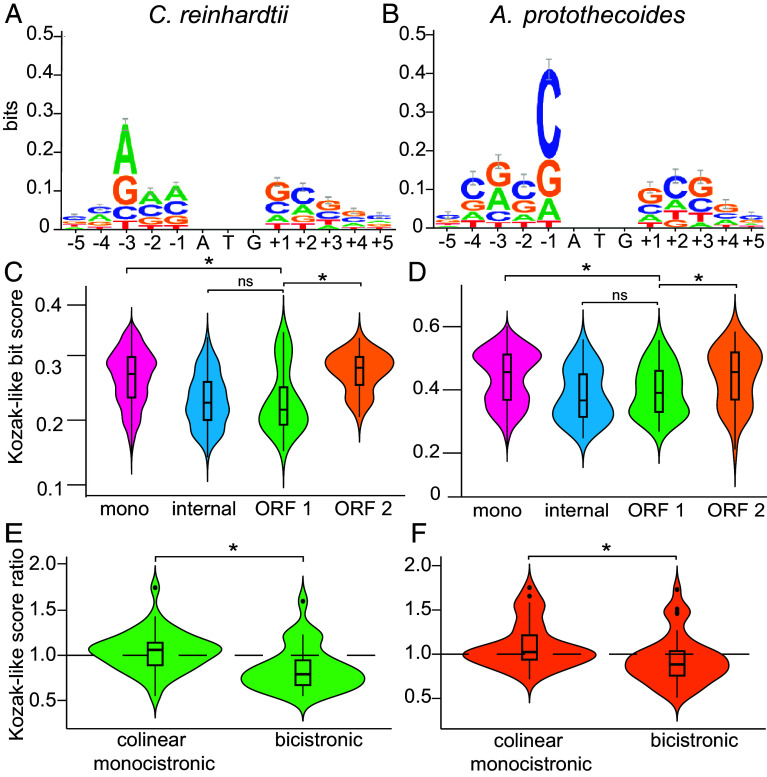
ORF 1 in bicistronic loci is associated with weak Kozak-like sequences. (*A*) WebLogo depicting the Kozak-like consensus sequence for *C. reinhardtii* based on a high-quality subset of annotated monocistronic genes (n = 7,210). (*B*) Kozak-like consensus sequence for *A. protothecoides* generated with a random subset of monocistronic genes (n = 2,598) (*C*) The distribution of Kozak-like sequence bit scores for *C. reinhardtii* bicistronic ORF 1 (n = 36), ORF 2 (n = 36), start codons upstream of ORF 2 excluding the annotated ORF 1 start codon (internal, n = 188), and the other half of monocistronic genes not used in the generation of the Kozak-like consensus (mono, n = 7,210). (*D*) The distribution of Kozak-like sequence bit scores for *A. protothecoides* bicistronic ORF 1 (n = 43), ORF 2 (n = 43), start codons upstream of ORF 2 excluding the annotated ORF 1 start codon (internal, n = 54), and monocistronic genes not used in the generation of the Kozak-like consensus (mono, n = 2,598). (*E* and *F*) Ratios of the bit scores of ORF 1 to ORF 2 were calculated for all bicistronic genes in *C. reinhardtii* (n = 36) and *A. protothecoides* (n = 43). Colinear monocistronic (n = 40) denotes a random set of adjacent genes on the same strand with ≤5,000 bp between ORFs. The dashed line denotes a bit score ratio of 1. Box plots depict the 25th, 50th, and 75th percentile. Whiskers indicate 1.5 times the interquartile range. Outliers are plotted as individual points. Statistical significance was determined with a Kruskal–Wallis test and Wilcoxon rank-sum test for sample comparison. Asterisks “*” indicate *P*-values less than 0.05.

The Kozak-like sequence determines the frequency with which a translation initiation site is recognized. In the context of leaky scanning, for translation of ORF 2 to occur, the Kozak-like sequence of ORF 1 should be suboptimal, allowing the preinitiation complex to occasionally scan through ORF 1. To evaluate this model, Kozak-like consensus sequences were deduced for both *C. reinhardtii* and *A. protothecoides* (Fig. 2 *A* and *B*). The Kozak-like consensus sequence varies between different eukaryotic lineages (38, 39) but is broadly defined as gcc(A/G)ccAUGG (3), with A or G at the −3 position and G at the +1 position relative to the initiator codon as the most conserved elements. Indeed, both algae retain these conserved features for monocistronic genes (Fig. 2 *A* and *B*), but the *A. protothecoides* consensus is more GC-biased than is the *C. reinhardtii* one, particularly at the −1 position relative to the initiator codon. Sequence logos of the ORF 2 initiation site in the bicistrons typically show conservation of the Kozak-like sequence and resemble the general consensus motif in each species (*SI Appendix*, Fig. S2). However, the ORF 1 initiation sites exhibit a weaker consensus and display a propensity for subpar Kozak-like sequences (*SI Appendix*, Fig. S2).

We used the consensus sequence to quantify the Kozak-like elements associated with individual ORFs. WebLogo density bit scores indicate how well each Kozak-like sequence resembles the deduced consensus (presumed optimal) sequence (40, 41). Bit scores were calculated for monocistronic, bicistronic ORF 1, and bicistronic ORF 2 genes based on five nucleotides upstream and downstream of the annotated initiation codons (Fig. 2 *C* and *D*). For each species, half of the monocistronic genes, chosen at random, were used to produce the original consensus sequence, while the remaining half were used to calculate the distribution of bit scores. We also extended this analysis to calculate the potential Kozak-like sequence scores associated with each of the AUGs upstream of the annotated ORF 2 start sites as they may serve as potential alternative start sites in case of a leaky scanning event (Fig. 2 *C* and *D*, denoted as “internal”). In both species, the scores for ORF 1 are significantly weaker than those for bicistronic ORF 2 and the monocistronic ORFs (Fig. 2 *C* and *D*). Scores for “internal” AUGs are also significantly weaker than for bicistronic ORF 2 and monocistronic ORFs (Fig. 2 *C* and *D*). This is compatible with a model in which ORF 1 of a bicistronic locus (and downstream alternative AUGs) permits intermittent leaky scanning. In contrast to ORF 1, ORF 2 would be expected to have a stronger Kozak-like sequence to maximize its translation. We tested this assumption by comparing the ratios of ORF 1 to ORF 2 Kozak-like bit scores for each individual bicistronic locus with the ratios of a randomly selected set of neighboring monocistronic genes (within 3,000 nt of each other). Given the propensity for a weak Kozak-like sequence at ORF 1 and a stronger Kozak-like sequence at ORF 2, we expected ratios of less than 1 for the bicistronic ORFs. Conversely, since mRNAs from adjacent genes are translated independently, the Kozak-like sequence of one transcript should have no influence on the other, and we expected ratios close to 1 for such monocistronic genes. In both organisms, the average ratio for the bicistronic genes was significantly smaller (median = 0.79 for *C. reinhardtii*, median = 0.88 for *A. protothecoides*) than for the monocistronic control sets (median = 1.06 for *C. reinhardtii*, median = 1.02 for *A. protothecoides*), indicating that the Kozak-like sequences of each ORF 1 are in a favorable context for occasional leaky ribosome scanning to occur while the ORF 2 Kozak-like sequence is favorable for consistent translation if ORF 1 is bypassed (Fig. 2 *E* and *F*).

### Bicistronic Transcripts Show AUG Depletion in Regions Before ORF 2.

The occurrence of AUGs upstream of the authentic initiation codon can inhibit translation of a downstream ORF (42–44). To test the frequency of alternative initiation codons in bicistronic loci, we calculated the observed frequency of “AUG” 3-mers within regions of the bicistronic transcripts and compared it to an expected estimation based on the sequence length (*Materials and Methods* and Dataset S4 A–P). The probability was adjusted based on the GC bias of the *A. protothecoides* and *C. reinhardtii* sequence elements to compare the expected frequency of “AUG” sequences in the 5′ UTR, ORF 1, the inter-ORF region, the total cumulative sequence upstream of ORF 2 (“before” ORF 2), ORF 2 of bicistrons, and monocistrons.

**Fig. 3.**
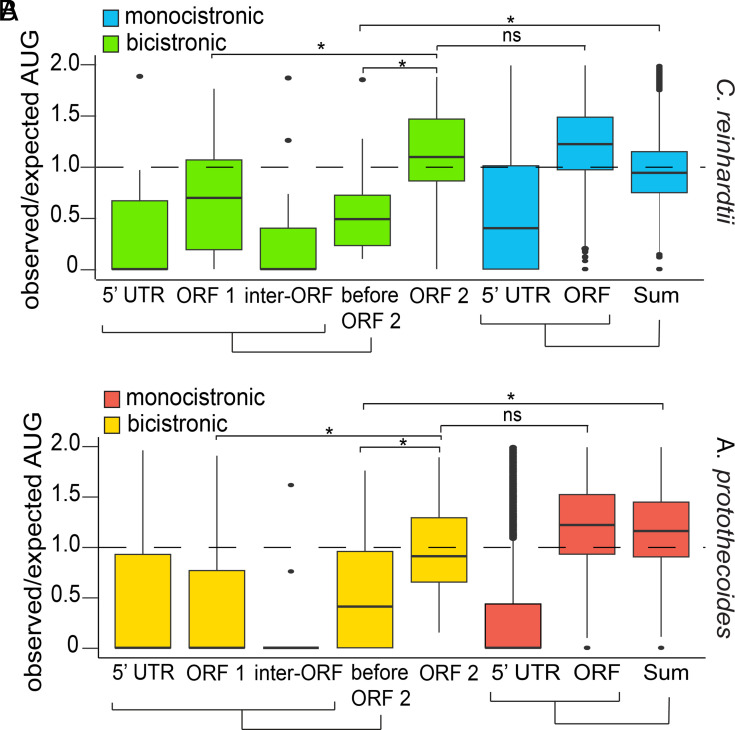
A bias against start codons upstream of ORF 2 in bicistronic loci. (*A* and *B*) Distribution of observed/expected ratios of “AUG” sequences based on nucleotide length. For the bicistronic (n = 36 & n = 43) set, this ratio was calculated for both individual components of the transcripts (“5′ UTR, “ORF 1”, “Inter-ORF”, “ORF 2”) and the sum of the components before the start codon of the downstream ORF (“before ORF 2”). For monocistronic genes (n = 15,333 & n = 7,698), the analysis was done for all annotated 5′ UTRs, ORFs, and the sum of those regions (Sum). Box plots depict the 25th, 50th, and 75th percentile. Whiskers indicate 1.5 times the interquartile range. Outliers are plotted as individual points. Statistical significance was determined with a Kruskal–Wallis test and Wilcoxon rank-sum test for sample comparison. Asterisks “*” indicated *P*-values less than 0.0001.

To assess the validity of this method for predicting the frequency of start codons, we calculated Pearson Correlation Coefficients (PCCs) between “AUG” frequencies within 5’ UTRs and ORFs, and their sequence lengths for monocistronic genes. There was a high positive correlation between the “AUG” count and the 5′ UTR plus ORF sequence length (*r* = 0.89 for *C. reinhardtii*, and *r* = 0.76 for *A. protothecoides*) for both algae, indicating that this was a valid metric for predicting “AUG” sequence bias (*SI Appendix*, Fig. S3). In theory, a sequence with no bias against “AUG” sequences should have an observed/expected ratio value of 1. Values less than 1 indicate bias against “AUG” sequences. The observed/expected values of the 5′ UTRs in monocistronic genes had median values of 0.47 and 0 in *C. reinhardtii* and *A. protothecoides*, respectively. Assuming correct annotation of the initiation codons, this bias against AUG sequences in the 5′ UTRs is consistent with maximizing ORF translation. Median observed/expected values for the monocistronic ORFs and for the combined 5′ UTR and ORF sequences ranged from 0.94 to above 1.0 in both algae (Fig. 3 *A* and *B* and Dataset S4), suggesting no discrimination against AUG 3-mers in these regions. Likewise, the observed/expected AUG ratios for ORF 2 of bicistrons also had medians near 1.0. However, we observed a striking reduction in observed/expected values of AUGs in ORF 1 in both algae, which were significantly lower than for ORF 2 (medians = 0.71 and 0 for *C. reinhardtii* and *A. protothecoides*, respectively). A bias against AUGs in the regions upstream of bicistronic ORF 2 was noted for both algae, with median values of 0.49 and 0.48 (Fig. 3 *A* and *B*). This bias against potential alternative start sites could suggest optimization for ribosome scanning to ensure correct translation of ORF 2. The relatively small size of bicistronic ORF 1 regions (*SI Appendix*, Fig. S1 *B* and *E*) may help to minimize the frequency of translation from other AUGs.

### Correlation of RNA and Ribosome Abundance in *C. reinhardtii*.

To better understand how bicistrons behave in vivo, we first turned to genome-wide expression patterns in the transcriptome and translatome of *C. reinhardtii*. Previous analyses have demonstrated that authentic polycistronic transcripts display nearly identical mRNA abundance estimates for each individual ORF (9, 28). To test this for our filtered set, we calculated PCC values for the bicistronic ORFs using log_10_ normalized reads from pooled transcriptomic (RNA-Seq) datasets (28). We further extended this analysis to log_10_ normalized reads from a ribosome profiling (Ribo-Seq) dataset (29) to compare ribosome occupancy at each ORF. For comparison, two control sets were assembled, one consisting of a random sample of colinear genes, and another set of randomly paired photosynthesis-related genes, as predicted by gene ontology (GO) annotation (45). The colinear genes showed correlation coefficient (*r*) values of 0.10 and 0.002 for the RNA-Seq and Ribo-Seq, indicative of negligible correlation as expected (*SI Appendix*, Fig. S4*B*). To ensure that this result did not occur from sampling bias, we repeated this analysis for the whole population of colinear genes, which showed a similar lack of correlation (*SI Appendix*, Fig. S4*A*). Although higher than what was seen in colinear genes, photosynthetic gene pairs still showed negligible correlation of read abundance and ribosome occupancy (*SI Appendix*, Fig. S4*B*, r = 0.24 and *r* = 0.15 for RNA-Seq and Ribo-Seq respectively). As previously observed (28), there is a very high positive correlation between ORF 1 and ORF 2 RNA-Seq read abundances in the bicistronic dataset (*r* = 0.92, *SI Appendix*, Fig. S4 *B*, *Top Right*). While not as highly correlated as the RNA-Seq abundances, a low positive correlation was observed in ribosome occupancy between ORF 1 and ORF 2 (*r* = 0.49, *SI Appendix*, Fig. S4 *B*, *Top Left*). Additionally, Ribo-Seq read abundance was almost always higher for ORF 1 than for ORF 2 (32/35 loci) (*SI Appendix*, Fig. S4 *B*, *Top Left*), implying lower levels of ORF 2 translation. This trend is consistent with observed Ribo-Seq patterns and proteomics studies in which alternative translation initiation events generate multiple ORFs (46–48). To assess the impact of Kozak-like sequences upstream of ORF 2 on its translation efficiency, we analyzed the relationship between the strength of that Kozak-like sequence and the ribosome footprint density. When there are strong Kozak-like sequences upstream of ORF 2, the ribosome footprint density is lower at ORF 2 compared to ORF 1 (*SI Appendix*, Fig. S5). The frequency of ORF 2 translation events is inversely correlated with the strength of Kozak-like sequences associated with upstream AUGs, consistent with the leaky ribosome scanning model.

### In Vivo Manipulation of ORF 1 Kozak-Like Sequence Influences Expression of ORF 2.

**Fig. 4.**
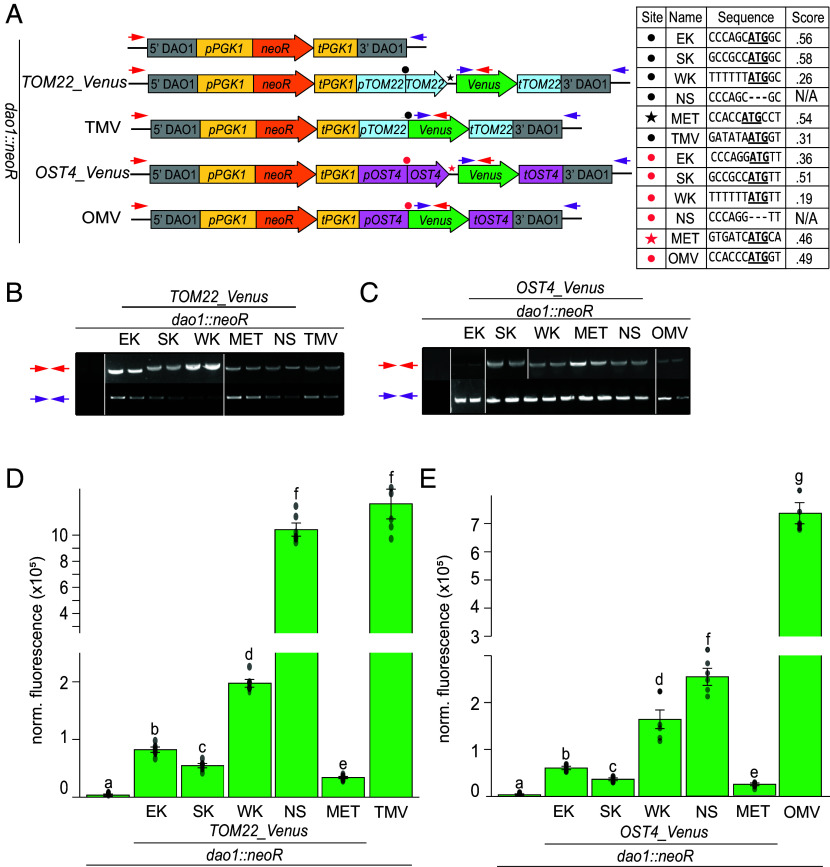
The Kozak-like sequence and start codon of ORF 1 influences ORF 2 expression in vivo. (*A*) Construct designs for targeted integration of a Venus reporter module into the *DAO1* neutral locus in *A. protothecoides*. Targeting 5′ and 3′ flanks are depicted in light gray. For G418 selection, the *neoR* expression module consists of a promoter sequence from *PGK1* (*pPGK1*) (yellow), the *neoR* ORF (orange), and a terminator sequence from *PGK1* (*tPGK1*) (yellow). Blue and pink *shading* indicate promoter (p), ORF, and terminator (t) regions from the *TOM22* and *OST4* bicistronic genes respectively. Lime-green shading represents the *Venus* ORF swapped into ORF 2. Inter-ORF regions are denoted by black lines separating the ORFs. Asterisks denote mutation sites, with specific sequences in the table. EK = Endogenous Kozak, SK = Strong Kozak, WK = Weak Kozak, NS = No start, TMV = *TOM22* Monocistronic *Venus*, OMV = *OST4* Monocistronic *Venus*. MET denotes a construct with the endogenous ORF 1 Kozak-like sequence and an additional out-of-frame ATG in the inter-ORF. Initiator codons are bolded and underlined. Dashes denote nucleotide deletions. The underscore (_) in construct names denotes the presence of the inter-ORF sequence in strains carrying bicistronic genes. Red and purple arrows denote the positions of genotyping primers. (*B* and *C*) Genomic PCR amplification of the *TOM22_Venus* and *OST4_Venus* cassettes at the *DAO1* locus (Dataset S6) (*D* and *E*) Venus fluorescence from strains expressing the *TOM22_Venus* and *OST4_Venus* constructs. Fluorescence for each strain was measured using six independent transformants. For normalization, background fluorescence was subtracted based on autofluorescence of simultaneously measured wild-type cells. The resulting values were then further normalized to the cell density (OD_750_) of each sample. For both *D* and *E*, Kruskal–Wallis and Wilcoxon rank-sum tests were applied to determine the statistical significance of sample comparisons. Letters indicate groups with comparative statistically significant differences (*P*-values less than 0.05).

Having established that the structural patterns, sequence properties, and expression correlations of bicistronic loci were compatible with the leaky scanning mechanism, we sought to further test the mechanism in vivo. For these experiments, gene targeting by homologous recombination in *A. protothecoides* was utilized to allow transgene insertions and mutational analyses with minimal positional effects. Quantifying the translation of endogenous bicistronic genes, many of which have unknown functions, posed a challenge; consequently, we designed a reporter system by replacing ORF 2 of endogenous bicistronic loci with the *Venus* ORF. These designs were applied to two such loci: *TOM22_SDHAF3* (UTEX250_A10.30135 and UTEX250_A10.30130) and *OST4_FAM32A* (UTEX250_A05.11125 and UTEX250_A05.11130) (*SI Appendix*, Fig. S6), selected on the basis of adequate mRNA expression for Venus detection, and conservation between *A. protothecoides* and *C. reinhardtii* (Dataset S1). These modified bicistronic genes were included in a cassette that also contained a selectable marker module conferring G418 antibiotic resistance and were integrated into the UTEX 250-A genome at a neutral homozygous landing site (*DAO1*) using targeting flanks (Fig. 4*A*).

To test whether the presence of ORF 1 influences ORF 2 expression, a construct was created in which the ORF 1 start codon was removed entirely (NS). Variant constructs were also designed in which the endogenous Kozak-like sequence (EK) of ORF 1 was mutated to be either stronger (SK) or weaker (WK) based on optimal and suboptimal Kozak-like sequences (Figs. 2*B* and 4*A*). Mutations were only introduced upstream of the initiation codon of ORF 1 to avoid altering the protein-coding sequence (Fig. 4*A*). To evaluate the influence of upstream alternative initiation codons on *Venus* translation, an additional construct was designed that preserved the endogenous ORF 1 Kozak-like sequence but contained an additional out-of-frame AUG in the inter-ORF region (MET). A construct that only contains the selectable marker module (*neoR*) targeted to *DAO1* provided a negative control, and monocistronic *Venus* driven by the endogenous promoters with the ORF 2 Kozak-like sequence (TMV and OMV) provided a positive control.

All strains were genotyped via PCR amplification of the gene cassette and neighboring sequence to ensure proper integration at the *DAO1* neutral landing site prior to phenotypic analysis (Fig. 4 *A* and *B*). Although precise integration of the constructs should minimize variability of reporter expression due to position effects, we measured the abundance of the bicistronic transcripts encoding *Venus* using RT-qPCR to test for significant differences in transcription or mRNA maintenance among the strain*s*. In both the *TOM22_Venus* and *OST4_Venus* contexts, there was no significant difference in transcript accumulation between strains (*SI Appendix*, Fig. S7), indicating that differences in Venus fluorescence between strains should be attributed to *Venus* translation rather than to RNA template abundance.

In both *TOM22_Venus* and *OST4_Venus* strains, Venus fluorescence was substantially higher than the background fluorescence detected in negative control lines containing only the selectable marker (*dao1::neoR*) (Fig. 4 *D* and *E*), but all bicistronic configurations with Venus in the ORF 2 position showed significantly less fluorescence than did the monocistronic Venus (*OMV*, *TMV*). The introduction of an out-of-frame AUG into the inter-ORF regions (MET) led to a significant decrease in Venus fluorescence compared to all other strains, with less than half the expression of EK strains (Fig. 4 *D* and *E*). These results indicate that alternative potential translation start sites upstream of ORF 2 with strong Kozak-like sequences limit ORF 2 translation, consistent with either a leaky-scanning or posttranslation-reinitiation model. The *TOM22_Venus* strain lacking the *TOM22* start codon (NS) had almost as much fluorescence signal as did the authentic monocistronic *Venus* strain (Fig. 4*D*). Although not as drastic, the removal of the ORF 1 initiator codon (NS) from *OST4_Venus* increased *Venus* fluorescence over fivefold in comparison to strains in which the integrity of ORF 1 is maintained (Fig. 4*E*). The higher expression in the *TOM22* background compared to *OST4* may be attributed to the lack of any internal AUG sequences in the *TOM22* coding sequence and inter-ORF regions, effectively making a monocistronic mRNA with an extended 5′ UTR. In contrast, the *OST4* coding sequence contains a single out of frame AUG codon with a weak Kozak-like sequence (bit score of 0.34), consistent with the trend for start site sequences upstream of ORF 2 (Fig. 2*D*, *SI Appendix*, Fig. S6, and Dataset S3). NS constructs are analogous to the most extreme case where there is no translation from the ORF 1 initiator codon. The *OST4_Venus* NS construct has higher Venus activity than does the corresponding WK construct, consistent with the leaky scanning hypothesis (reduced translation upstream of ORF 2 leads to increased ORF 2 translation), and inconsistent with the reinitiation model (translation upstream of ORF 2 and ORF 2 translation are positively correlated). Furthermore, increased Venus translation on removal of the ORF 1 initiation codon argues against the involvement of autonomous elements such as IRESs in ORF 2 translation (in which situation ORF 2 translation would be independent of ORF 1 translation).

Next, we examined the effects of altering the ORF 1 Kozak-like sequence, hypothesizing that an inverse relationship between the strength of the ORF 1 Kozak-like sequence and *Venus* fluorescence would be consistent with a leaky scanning mechanism. Fluorescence decreased by factors of 1.5 and 1.7, in strains expressing *TOM2_Venus* and *OST4_Venus*, respectively, with a stronger ORF 1 Kozak-like sequence (SK) compared to the endogenous Kozak-like sequence (EK) (Fig. 4 *D* and *E*). In contrast, weaker Kozak-like sequences (WK) increased fluorescence over twofold in both contexts (Fig. 4 *D* and *E*). The inverse correlation between the strength of the Kozak-like sequence of ORF 1 and downstream *Venus* translation is consistent with leaky scanning and would not be expected if reinitiation were the main driving force for ORF 2 translation.

### A Synthetic Bicistronic Locus Displays Coexpression and Tunability via the Kozak-Like Sequence.

**Fig. 5.**
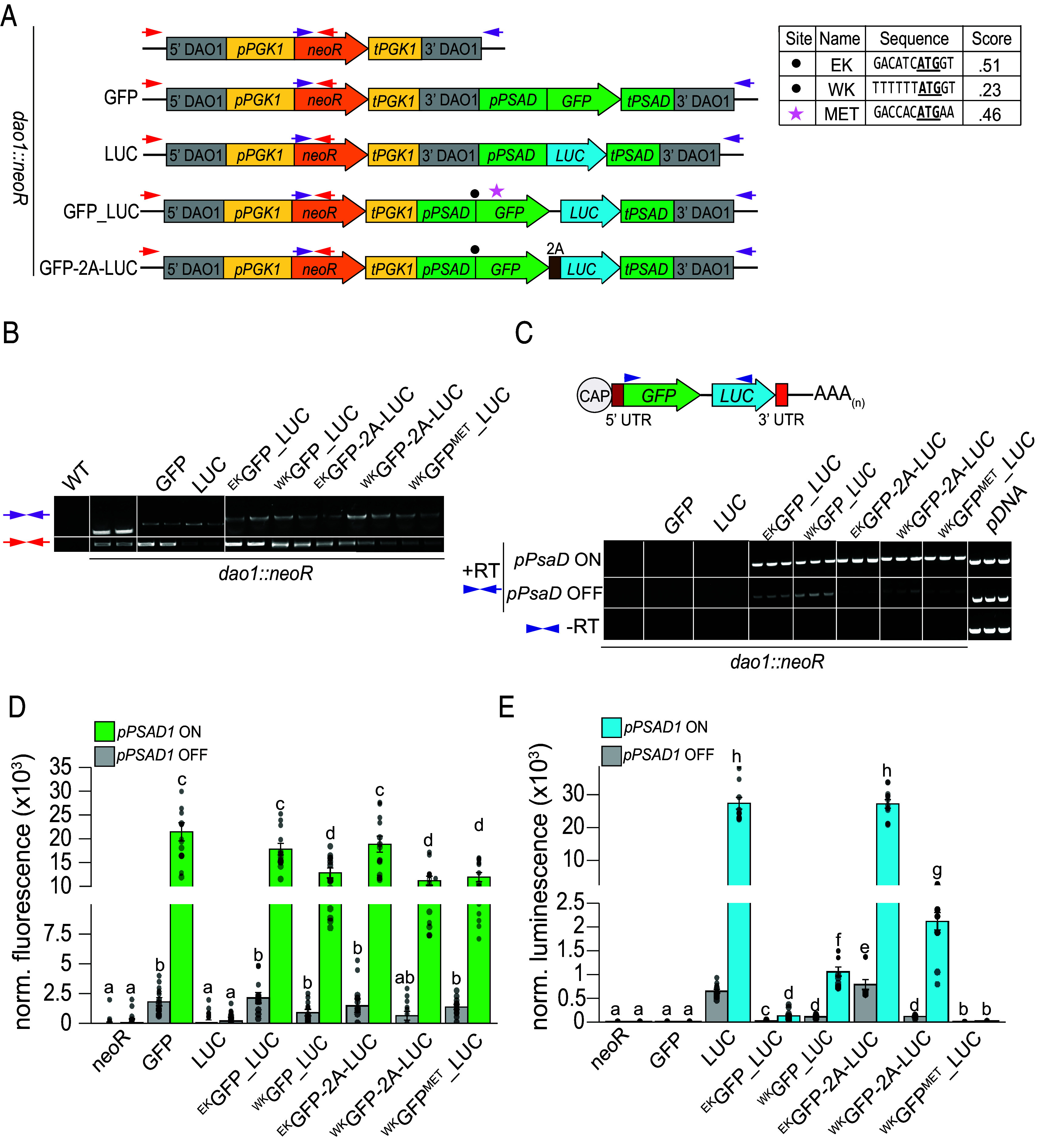
A synthetic bicistronic transcript displays tunable activity via the strength of the Kozak-like sequence. (*A*) Construct designs for a synthetic bicistronic dual reporter in *A. protothecoides*. *DAO1* targeting flanks and G418 resistance components are the same as previously described in Fig. 4*A*. The *REX1* inter-ORF is denoted as the black line between the *GFP* and *LUC* ORFs. Bicistronic expression of SuperFolder *GFP* (lime-green) and *Gaussia princeps* Luciferase (*LUC*) (blue) were driven by the *PSAD1* promoter (*pPSAD*) (dark green). Black circles denote Kozak mutation sites, with specific sequences shown in the table. EK = Endogenous Kozak, WK = Weak Kozak. MET denotes a construct with the reintroduction of an AUG codon into the ORF of the *GFP* (pink star). The underscore (_) in construct names denotes the presence of the *REX1* inter-ORF. Red and purple arrows indicate the positions of primers used for genotyping. (*B*) Genomic PCR amplification verifying integration of constructs at the *DAO1* locus (Dataset S6). Results from two representative transformants are shown for each strain. (*C*) RT-PCR of synthetic bicistronic mRNAs. The positions of primers for amplification of both ORFs are indicated on the schematic with blue arrows (Dataset S6). RT indicates the presence (+) or absence (−) of reverse transcriptase. Results from three representative transformants are shown for each strain. (*D*) Normalized GFP fluorescence from strains grown under heterotrophic (gray) or photoautotrophic (green) conditions. (*E*) Normalized luminescence from strains grown under heterotrophic (gray) or photoautotrophic (blue) conditions. Fluorescence and luminescence for each strain were measured using 12 independent transformants. For normalization, background noise was subtracted based on the value of simultaneously measured wild-type cells. The resulting values were further normalized by cell density (OD_750_ of each sample). For both *D* and *E*, Kruskal–Wallis and Wilcoxon rank-sum tests were applied to determine the statistical significance of sample comparisons. Letters indicate comparisons with statistically significant differences (*P*-values less than 0.05).

In light of the observation that the ORF 1 Kozak-like sequence influences ORF 2 translation in vivo, we designed a synthetic bicistronic dual reporter to quantify the coexpression of both transgenes and evaluate the effect of Kozak-like sequence manipulation. For high levels of transcription, we utilized the promoter and terminator regions from the gene encoding photosystem I subunit D (*PSAD1)*, which in *A. protothecoides* is induced in photoautotrophy and repressed in heterotrophy. Consequently, we could leverage the reversible trophic switch to inhibit or induce expression (Fig. 5*A*). Conveniently, the predicted size of the *PSAD1* 5’ UTR is also close in size (46 nt) to the median length of 5′ UTRs of endogenous bicistronic transcripts (*SI Appendix*, Fig. S1). The *PSAD1* Kozak-like region is close to the consensus, maintaining the highly conserved elements at the −3 and −1 positions (Fig. 5*A*).

In the ORF 1 position, we incorporated a codon-optimized SuperFolder *GFP* (49) that was modified to replace five codons encoding methionine with leucine (50), ensuring that there are no potential in- or out-of-frame initiation codons within the ORF. In the ORF 2 position, we placed a reporter gene encoding *Gaussia princeps* luciferase (*LUC*) fused to a signal peptide from an *A. protothecoides* secreted carbonic anhydrase (CAH1) (51). The ORFs were separated by the 14 nt inter-ORF sequence from the endogenous *REX1S_REX1B* bicistronic locus (UTEX250_A11.30960 and UTEX250_A11.30955). We then tested the effects of maintaining (^EK^*GFP_LUC*) or weakening (^WK^*GFP_LUC*) the strong *PSAD1* Kozak-like sequence in bicistronic reporters. A bicistronic construct with a weak ORF 1 Kozak-like sequence and a single methionine codon reintroduced into the *GFP* ORF (^WK^*GFP*^MET^_*LUC*) was used to test the effect of alternative upstream translation initiation sites on ORF 2 expression. This reintroduced AUG was deliberately chosen due to its relatively high Kozak-like score (Fig. 5*A*). Positive control strains expressed monocistronic *GFP* or *LUC* driven by the *PSAD1* promoter with the endogenous Kozak-like sequence (Fig. 5*A*). For comparison, we also included strains in which the stop codon of *GFP* was removed and the inter-ORF sequence was swapped with a 2A self-cleaving peptide sequence, so that *GFP* and *LUC* were translated from a single ORF. This was conducted in the context of the endogenous (^EK^*GFP-2A-LUC*) and weak (^WK^*GFP-2A-LUC*) Kozak-like sequences, serving as comparisons for coexpression.

Transformed strains were genotyped via PCR amplification to ensure precise insertion at the *DAO1* landing site (Fig. 5 *A* and *B*, primers illustrated by red and purple arrows). Transgene mRNAs were detected by RT-PCR in all lines using primers spanning the *GFP* and *LUC* ORFs (Fig. 5*C*). Transcripts of the expected sizes were present in all dual reporter strains, and were absent in negative (*neoR*) and monocistronic positive controls (*GFP* and *LUC*). RT-PCR products with *GFP* and *LUC* separated by either inter-ORF or 2A sequences were confirmed by sequencing (*SI Appendix*, Fig. S8). No amplification products were detected in controls run without reverse transcriptase, confirming the absence of gDNA contamination (Fig. 5*C*). Transcript abundance was substantially reduced in heterotrophy compared to photoautotrophy in all strains, consistent with control of transgene mRNA accumulation by *PSAD1* regulatory sequences (Fig. 5*C*).

RT-qPCR was conducted using ORF-specific primers for *GFP* and *LUC* to test for differences in RNA abundance in strains expressing the reporter constructs (*SI Appendix*, Fig. S9). No significant differences in transgene expression were observed between strains grown under photoautotrophic conditions, indicating that variations in GFP and luciferase activity were due to differential translation rather than transcript abundance. However, both GFP and luciferase reporter activities were considerably higher in photoautotrophic compared to heterotrophic cells (Fig. 5 *D* and *E*), consistent with increased transgene expression (Fig. 5*C*). GFP fluorescence and luciferase activities were detected in all strains with bicistronic expression. As expected, the activities of both reporters were positively correlated in response to Kozak-like sequence strength in strains expressing GFP and LUC from a single ORF linked by a 2A peptide. GFP fluorescence was reduced by twofold and luminescence was reduced by 10-fold in ^WK^*GFP-2A-LUC* strains compared to monocistronic controls (*GFP* and *LUC*) and ^EK^*GFP-2A-LUC* strains, consistent with reduced translation of the *GFP-2A-LUC* ORF (Fig. 5 *D* and *E*). The more dramatic decrease in luciferase activity compared to GFP fluorescence in the ^WK^*GFP-2A-LUC* strain could be attributed to inefficient cleavage of the 2A peptide impeding luciferase protein function or secretion. The 2A peptide sequence does not contain any alternative start sites or internal ORFs; hence both translation and processing of GFP-2A-LUC and leaky ribosome scanning could contribute to *LUC* translation. This feature may explain the higher levels of luciferase activity in ^WK^*GFP-2A-LUC* compared to ^WK^*GFP-LUC* strains (Fig. 5*E*). Conversely, manipulating the ORF 1 Kozak-like element in bicistronic dual reporter constructs resulted in a negative correlation between GFP and LUC activity. In all strains expressing *GFP* with a weak Kozak-like sequence (^WK^*GFP*), GFP fluorescence decreased significantly in comparison to strains with endogenous Kozak-like sequences (^EK^*GFP*) (Fig. 5*D*). Conversely, luciferase activity increased nearly 10-fold in ^WK^*GFP_LUC* strains compared to ^EK^*GFP_LUC* strains, demonstrating the inverse relationship between ORF 1 and ORF 2 translation consistent with leaky scanning (Fig. 5*E*). Strikingly, ^WK^*GFP*^MET^*_LUC* strains had far less luciferase activity compared to the strains lacking the internal methionine (Fig. 5*C*). This was likely a result of reduced translation of ORF 2 *LUC* due to the presence of an alternative initiation site with a strong Kozak-like sequence within the *GFP* ORF (Fig. 5*E*), which would inhibit ribosomes from reaching the luciferase start codon. GFP fluorescence and luciferase luminescence were visualized for further validation of expression (*SI Appendix*, Fig. S10).

## Discussion

### Evidence for Leaky Scanning as the Mechanism for Multiple ORF Translation.

In this study, we tested multiple hypotheses to identify the primary mechanism for translation of multiple ORFs in bicistronic transcripts in green algae. Based on previous analyses (28), leaky scanning and posttermination reinitiation were identified as potential mechanisms driving translation of multiple ORFs. These mechanisms are not mutually exclusive (52), and while we cannot completely discount some degree of reinitiation, there is robust evidence that supports the leaky scanning model as the primary driver for downstream ORF translation. Bioinformatic and structural analyses of high-confidence *C. reinhardtii* and *A. protothecoides* bicistronic loci revealed a prevalence of short 5′ UTRs (*SI Appendix*, Fig. S1*A*), and weak Kozak-like sequences at the translation initiation site of ORF 1 (Fig. 2), features that promote intermittent leaky scanning. Indeed, in vivo mutational studies demonstrated that ORF 2 translation is inversely correlated with the strength of the Kozak-like sequence of ORF 1, both in the context of two endogenous bicistronic loci (Fig. 4) and a synthetic *GFP_LUC* bicistron (Fig. 5). A similar result was also seen in Kozak-like sequence manipulations of *C. zofingiensis* bicistronic mRNAs in vitro (28). The opposite result would be expected if reinitiation were the primary mechanism, i.e. Kozak-manipulation would generate a positive correlation between translation of ORF 1 and reinitiation at ORF 2.

We also observed reduced occurrence of AUGs upstream of ORF 2 (Fig. 3). Since posttermination 40S ribosomes can move bidirectionally for short distances and reinitiate at upstream or downstream AUGs (22), underrepresentation of AUG codons in ORF 1 and the inter-ORF regions (which are generally short) could be explained in part by natural selection under the reinitiation model. Competition with AUG sequences close to the ORF 1 stop codon would be expected to reduce translation from the ORF 2 initiation site and would consequently be selected against. Alternatively, the dearth of internal AUG codons and their association, when present, with weak Kozak-like sequences (Fig. 3), is also consistent with adaptation to allow ribosomes to bypass the upstream regions and reach the ORF 2 initiation codon.

**Fig. 6.**
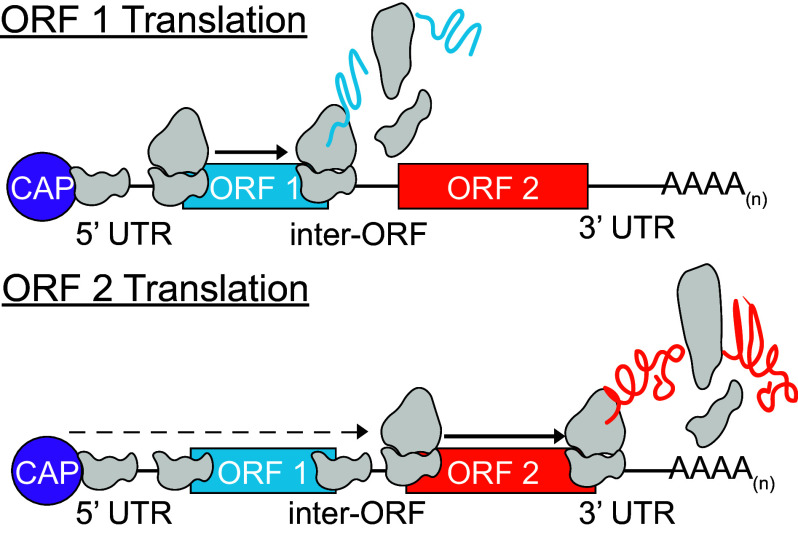
The mechanism driving bicistronic expression in green algae. Translation initiates at ORF 1 via cap-dependent scanning or a suboptimal ORF 1 Kozak-like sequence allows the preinitiation complex to bypass ORF 1 and translate ORF 2.

Reintroduction of an in-frame AUG in the *GFP* coding sequence of the *GFP_LUC* dual reporter severely diminished downstream luciferase activity (Fig. 5). The restored AUG was 483 bases upstream of the translation stop codon, likely beyond the limit for bidirectional reinitiation after termination of *GFP* translation, and so should not compete with the ORF 2 *LUC* initiation codon in the reinitiation model. The reintroduced AUG was also associated with a strong Kozak-like sequence, in principle enabling efficient translation of an N-terminally truncated GFP. If ORF 2 translation was governed by reinitiation, we would expect this alternative initiation site to stimulate increased *LUC* translation: Instead, the opposite was observed. It is therefore more likely that the reincorporated AUG competed for ribosomes that scanned past the authentic AUG of ORF 1 and initiated instead at the reintroduced AUG, thereby reducing ORF 2 *LUC* translation. These findings, taken collectively, support a model in which green algal bicistronic expression occurs via cap-dependent initiation of ORF 1, coupled with episodic leaky ribosome scanning that allows ORF 2 translation (Fig. 6). Similar observations were reported in an earlier study describing *stoned* and *Snapin*, two bicistronic loci in *Drosophila melanogaster* (53). The upstream ORFs at both Drosophila loci are deficient in internal AUG codons, and the Kozak-like regions are diverged from the consensus (53). The in vivo mutational analysis in the Drosophila system using bicistronic reporter constructs also indicated that translation of the downstream ORF was dependent on leaky ribosome scanning (53).

Previous efforts at bicistronic transgene expression in green algae have had limited success, and it has been difficult to test hypotheses as to the underlying mechanism. Onishi and Pringle (54) found that recovery of *C. reinhardtii* transformants with high expression of fluorescent reporters and fusion proteins was improved using bicistronic constructs with a reporter gene at ORF 1 and the *aphVIII* selection marker at ORF 2, separated by a six nt spacer sequence (54). While multiple different reporters were expressed successfully at the ORF 1 position, *aphVIII* was the only selectable marker that could be expressed at ORF 2. The authors postulated that ribosomal reinitiation was the most likely mechanism, but acknowledged inconsistencies with this model, specifically a documented negative correlation between uORF length and reinitiation efficiency, with the best efficiencies seen in short uORFs of only a few codons (52, 54, 55). Jacobebbinghaus et al. (56) integrated a series of transgenic constructs expressing bicistronic mRNAs in *C. reinhardtii* and observed that the different inter-ORF sequences alone produced dramatic differences in expression of both ORF 1 and ORF 2, as judged by fluorescence activity and transformation efficiency, respectively (56).

In light of these findings, the observations described by Onishi and Pringle (54) and Jacobebbinghaus et al. (56) are consistent with some contribution from leaky ribosome scanning to bicistronic gene expression in their systems. The bicistronic transgene constructs analyzed by Jacobebbinghaus et al. contained multiple AUG 3-mers within ORF 1 reporter and fusion genes, features which we expect would inhibit ORF 2 translation. We observed that, in general, the constructs analyzed by Jacobebbinghaus et al. that incorporated inter-ORF sequences with fewer AUG 3-mers in the inter-ORF region had higher relative transformation efficiency than those with a greater number of AUG 3-mers (*SI Appendix*, Fig. S11). Mechanistic interpretations of these results are confounded by position effects caused by random integration of the test constructs into the *C. reinhardtii* genome, and the use of multiple different inter-ORF sequences, each of which changes the Kozak-like sequence governing translation of the selectable marker gene at ORF 2. We controlled for these variables by uncoupling reporter and selectable marker gene expression, targeting all constructs to the same (homozygous) neutral landing site to reduce position effects, and minimizing alternative initiation codons upstream of ORF 2.

### Regulation of Bicistronic Gene Expression.

The ability of the 43S preinitiation complex to recognize a start codon within the proper sequence context is influenced by eukaryotic initiation factor 1 (eIF1), whose binding in the ribosomal P site promotes scanning and skipping of start sites with subpar Kozak sequences (57, 58). The translational landscape is profoundly altered in *eIF1* knockdown and knockout human cell lines, with ribosome footprints that reveal far less discrimination between canonical translation start sites and alternative upstream initiation sites with subpar Kozak-like sequences (59). Intracellular eIF1 concentrations are influenced by stress, suggesting a mechanism for regulating ORF expression ratios from algal bicistronic transcripts (60). In addition to the Kozak-like sequence, secondary structures around the initiation site promote leaky scanning and the presence (or lack thereof) specific secondary structures proximal to the ORF initiation site may also influence the rate of leaky scanning (61).

The *A. thaliana CDC26*_*TTM3* bicistron is conserved in both plants and green algae, and illustrates the interplay between nonsense-mediated decay (NMD) and translation (11, 62). *CDC26_TTM3* transcript levels were higher in *A. thaliana* NMD mutants compared to wild-type, suggesting NMD regulation, triggered by the short *CDC26* ORF (11). Conversely, translation of the *TTM3* ORF increased CDC26 abundance by enhancing ribosome recruitment to the bicistronic transcript, suggesting a mechanism for bypassing NMD to improve production of small proteins (11).

### Why Leaky Ribosome Scanning for Bicistronic Expression?

Polycistronic gene arrangements increase physical compactness of the genome and guarantee coordinated gene expression. Leaky ribosome scanning is a commonly used mechanism for translating viral polycistronic transcripts (20). Given genome size constraints, it is understandable that viruses employ leaky scanning for coordinated protein synthesis, but even the smallest green algal genomes are orders of magnitude larger than those of viruses. Alternatively, leaky scanning can contribute to dual functionality of a transcript via production of alternative protein isoforms. A suboptimal Kozak-like context sequence around the first AUG of the *S. cerevisiae PiF1* transcript, encoding a helicase, permits leaky scanning, resulting in a truncated protein isoform that is targeted to the nucleus instead of the mitochondrion (63). In *A. thaliana*, leaky scanning of the DNA polymerase-encoding *POLγ2* mRNA similarly generates alternative protein isoforms with targeting to either the chloroplast or the mitochondrion (64). Although some algal bicistronic transcripts have splice variants (28), we have not encountered examples encoding protein variants with alternative organellar targeting signals. Instead, some bicistronic genes may coexpress proteins that function together. However, this does not appear to hold for a majority of bicistrons, as functional predictions suggest that many encode pairs of proteins with different localizations and unrelated functions (Dataset S5 A and B). About a quarter to a third of proteins encoded at bicistronic loci are predicted to be localized to either the chloroplast or mitochondrion (*SI Appendix*, Fig. S12 and Dataset S5 C and D). This observation may suggest an ancestral plastid or mitochondrial origin of these nuclear genes: However, future studies are needed to test this idea.

uORFs are ubiquitous throughout eukaryotic genomes (65). Nearly 67% of annotated *C. reinhardtii* transcripts contain at least one uORF (40); they are present in more than half of the annotated transcripts in humans and mice (66) and 30 to 70% of plant mRNAs (48, 67). Their primary role appears to be inhibition of 5′ cap-dependent translation of canonical protein-coding ORFs (42, 66), where translation of canonical ORFs can proceed through alternative translation initiation events. The functional polypeptide encoding capacity of uORFs remains contentious, but it is evident that bicistronic transcripts in green algae occur as a continuum with the phenomenon of uORFs. The first ORF in green algal bicistronic genes can be considered to be a “large” protein-coding uORF, and like other uORFs, inhibits the translation of downstream ORFs (Figs. 4–6). Outside of this putative regulatory role, most algal ORF 1s are detected in proteomic experiments (*SI Appendix*, Fig. S12*A*) and are highly conserved within the green algal lineage, indicating that they are actually translated and function as proteins.

### The Functional Roles of Proteins from Bicistronic Loci.

To better determine the biological significance of proteins encoded by bicistronic transcripts, functional and localization predictions were performed. Over one-fourth and one-seventh of proteins encoded by bicistronic loci in *A. protothecoides* and *C. reinhardtii* respectively, are predicted to have localization or function associated with the mitochondria (*SI Appendix*, Fig. S12*C* and Dataset S5). UTEX250_A12.36865, an ortholog of human PET100, functions in the biogenesis of Complex IV and is a component of the mitochondrial electron transport chain (68). About 20% of the bicistronic genes in *A. protothecoides* and 10% in *C. reinhardtii* are predicted to encode proteins that function and localize to the nucleus. Notably, the *REX1S-REX1B* locus, which has been experimentally analyzed in *C. reinhardtii*, produces a bicistronic mRNA encoding two polypeptide subunits thought to function in DNA repair (69). Despite evidence of their conservation and translation, many bicistronic genes encode proteins of unknown function, with 29 predicted ORFs in *A. protothecoides* and 16 in *C. reinhardtii* having no functional predictions or known orthologs outside of the algae (*SI Appendix*, Fig. S12*B* and Dataset S5). These “pioneer” proteins present candidates for future reverse genetic analysis for novel gene discovery.

### Application and Utility in Synthetic Biology.

Green algae are promising microbial systems for synthetic biology and environmental applications such as bioremediation (27). Engineering in these systems requires expression of multiple transgenes to build synthetic biochemical pathways, and polycistronic arrangements may be useful for coordinating gene expression and modulating protein stoichiometry. Tunable coexpression of three ORFs and successful production of monoclonal antibody components was achieved in transient assays in human cell lines by modifying the order and the strength of the Kozak-like sequence for each ORF (50). Additionally, the recruitment of polysomes at bicistronic loci (11) could be of particular interest for improving the translation of small polypeptides that might otherwise be difficult to produce. As the process of translation initiation is widely conserved in eukaryotes, this mechanism of polycistronic expression may be widely applicable. That said, this strategy should employ multiple design considerations, including a short 5′ UTR, removal or limiting AUG sequences upstream of ORF 2, a suboptimal Kozak-like sequence at ORF 1, and a strong Kozak-like sequence at ORF 2. Outside of the context of polycistronic expression, manipulation of the Kozak-like sequence is a powerful tool for adjusting protein output without changing transcript abundance. In rabbits, manipulation of the Kozak-like sequence context of the *PCSK9* gene involved in cholesterol homeostasis induced phenotypic variations without influencing transcription (70). These insights can guide construct design for transgene expression and encourage studies to further optimize expression of multiple ORFs for use in synthetic biology.

## Materials and Methods

Bicistronic loci were identified in *C. reinhardtii* and *A. protothecoides* using RNA-Seq and Iso-Seq data, with further curation based on conservation and Ribo-Seq reads (*C. reinhardtii* only). The bicistrons and monocistronic controls were further analyzed based on their Kozak-like bit scores (based on a genome-wide consensus WebLogo) of annotated ORF start sites and “AUG” 3-mer counts. In vivo mutational analyses were performed in the *A. protothecoides* background strain UTEX 250-A via targeted integration of a gene cassette using a lithium acetate protocol. Transformants were genotyped to confirm proper integration and subsequently analyzed using fluorescence and luminescence assays. A detailed description of all methods and materials used in this study is provided in *SI Appendix*, *Materials and Methods*.

## Supplementary Material

Appendix 01 (PDF)

Dataset S01 (XLSX)

Dataset S02 (XLSX)

Dataset S03 (XLSX)

Dataset S04 (XLSX)

Dataset S05 (XLSX)

Dataset S06 (XLSX)

## Data Availability

*A. protothecoides* genome (33) assembly and annotations can be found at the US NCBI short read archive (SRA) under BioProject PRJNA1195245 for the genome and PRJNA1211023 for the Iso-Seq reads. The *C. reinhardtii CC-4532 v6.1* genome (71) can be accessed at Phytozome.net (https://phytozome-next.jgi.doe.gov/info/CreinhardtiiCC_4532_v6_1) or https://doi.org/10.1093/plcell/koac347 with Iso-Seq reads under the US NCBI SRA accession PRJNA670202. Further details for the *A. protothecoides* genome can be found at https://doi.org/10.1101/2025.02.07.637104.
