# Supplementary material for: Leaky ribosomal scanning enables tunable translation of bicistronic ORFs in green algae
Source: Proc Natl Acad Sci U S A. 2025 Feb 26;122(9):e2417695122. doi: 10.1073/pnas.2417695122 (PMC11892635; doi:10.1073/pnas.2417695122)
Supplement: Supplementary file 1 — Appendix 01 (PDF) [file pnas.2417695122.sapp.pdf]

# Supporting Information for
Leaky ribosomal scanning enables tunable translation of bicistronic ORFs in green algae.

Marco A. Dueñas[1], Rory J. Craig[2], Sean D. Gallaher[2,3], Jeffrey L. Moseley[2], Sabeeha S. Merchant[1,2,3,4]

Corresponding authors: Sabeeha S. Merchant, Jeffrey L. Moseley
Email: sabeeha@berkeley.edu, jlmoseley@berkeley.edu

**This PDF file includes:**

Supplementary text
Materials and Methods
Figures S1 to S13
Legends for Datasets S01 to S06
SI References

## Supplementary Results

**The inter-ORFs of bicistrons in *Auxenochlorella protothecoides* are unlikely to have IRES-like function**

One consideration is the possibility that bicistronic transcript inter-ORF regions contain “IRES- like” elements that directly recruit ribosomes for translation of the downstream ORF. In our previous analysis, IRESfinder predicted low scores for each of the inter-ORF sequences from the bicistronic loci of *Chlamydomonas reinhardtii* and *Chromochloris zofingiensis* (1). Upon repeating this analysis in *A. protothecoides*, IRESfinder prediction software found that, on average, the bicistronic inter-ORF sequences had significantly lower probability of containing IRESs than did sequences determined empirically to be IRESs and were not significantly different from random, size-matched intergenic sequences from the genome. (Supplementary Figure 13).

Our own observations also argue against IRES-like features in green algal bicistronic genes. Many algal inter-ORF regions are less than 10 nt long, with no evidence of conserved sequence motifs or structures, and some *A. protothecoides* loci even contain overlapping ORF 1 stop and ORF 2 start codons (Supplementary Dataset S01B). If inter-ORF regions had IRES-like properties, ORF 2 translation would be expected to be independent of cap-dependent translation initiation, and therefore, modifications to the ORF 1 Kozak-like sequence should not affect ORF 2 translation. However, our *in vivo* mutational analyses of endogenous and synthetic bicistronic loci demonstrated that ORF 2 translation was directly influenced by manipulation of sequence at the ORF 1 initiation site (Figures 4 and 5), contradicting the IRES hypothesis. These arguments support the notion that the inter-ORF sequences most likely do not contain IRES-like elements, but rather 5’ UTR length, strength of Kozak-like sequences, and the frequency of alternative translation initiation sites upstream of ORF 2 determine the amount of leaky scanning and ability of the ribosome to reach ORF 2.

## *Materials and Methods*

**Manual curation of bicistronic genes**

The transcriptomes and genomes of *A. protothecoides* (UTEX 250-A *v1.1*) (2) and *C. reinhardtii* (*CC-4532 v6.1*) were visualized on the Integrative Genomics Viewer (3) with input from Iso-Seq and RNA-Seq data. For *C. reinhardtii*, additional datasets for H3K4me3 ChIP-Seq, Ribo-Seq, and genome sequences for field strains CC-1952 and CC-2931 were included. Bicistronic loci for both algae were characterized in terms of 5' UTR size, ORF size, ORF spacing, co-expression relative to colinear genes, and conservation in other Chlorophyte species. A detailed description of the high-confidence evaluation and gene structure properties can be found in Supplementary Dataset S01 (S01A & S01B).

**Bicistronic loci conservation analysis**

Criteria for conservation was adapted from our previous work with minor modifications (3). The protein sequences encoded by all of the ORFs in the refined sets of bicistronic genes were used to search for orthologs in other green algae. Genomes were available from either Phytozome.net (*C. reinhardtii* v6.1, *C. zofingiensis v5.2.3.2, Coccomyxa subellipsoidea* C-169 v2.0, *Dunaliella salina* v1.0, *Ostreococcus lucimarinus* v2.0, *Micromonas pusilla* CCMP1545 v3.0, and *Volvox carteri* v2.1), Phycocosm.net (*Chlorella sorokiniana* UTEX 1602, *Chlorella vulgaris*, and *Nannochloris desiccata* UTEX 2437), GenBank (*Prototheca cutis*, accession BCIH01000000), or NCBI Short Read Archive (*A. protothecoides*, BioProject PRJNA1195245) (4). Orthologs were identified using blastp or tblastn bit score cutoffs of ≥30. The nuclear genomes were then queried to identify pairs of colinear ORFs (ORFs on the same on the same strand of the same chromosome separated by <10 kbp) as possible bicistronic loci. The list of candidates was then manually curated using either the JBrowse tool from Phytozome.net or visualization in Geneious Prime. When available, expressed sequence tag (EST) data from Phytozome was used to identify transcripts spanning two or more ORFs in candidate polycistronic loci. Similarly, Iso-Seq reads from *C. reinhardtii*, *C. zofingiensis*, *D. salina*, and *C. sorokiniana* were used to determine if colinear ORFs were transcribed as bicistronic mRNAs in those species. Additionally, some bicistronic orthologs in *C. reinhardtii* were identified by finding orthologs of newly identified *A. protothecoides* bicistronic proteins using the OrthoFinder software (5). These orthologs were confirmed manually as authentic bicistronic loci in IGV. For each high scoring pair of orthologs in each species, the protein ID, the coordinates of the gene encoding that protein in the corresponding genome assembly, the BIT score of the match, supporting evidence (either colinear ORFs, EST support, or Iso-Seq support) and the BLAST algorithm used can be found in Supplementary Dataset SO2 (S02A & S02B).

**Kozak-like sequence generation and Kozak bit score comparison**

Kozak scores for monocistronic and bicistronic genes were quantified using previously described methods (6,7). For *C. reinhardtii*, monocistronic nuclear gene models from the v6.1 annotation were first filtered to remove transposable element genes and any genes that did have at least one homolog via OrthoFinder in the previously described collection of Chlorophyceae and Trebouxiophyceae genomes. Next, the filtered monocistronic gene set (*n* = 14,738) was randomly divided into two datasets each containing equal numbers of genes. One set was used to generate a consensus Kozak logo using WebLogo 3 by extracting the 5 bp upstream and downstream of each annotated start codon. A Kozak score was then calculated for each of the genes in the second half of the monocistronic dataset, and for all the bicistronic genes. In the consensus logo, each of the four nucleotides at each site in the Kozak sequence has an associated bit score. The Kozak score is the sum of bit scores for each nucleotide in a query sequence based on the corresponding nucleotide in the consensus logo. The same method was applied to *A. protothecoides* strain UTEX 250-A. Nuclear monocistronic genes were filtered to remove genes without homologs to ensure authenticity of the annotation, and the haplotype A gene model was arbitrarily selected when a gene was represented by two alleles in the diploid genome. The filtered dataset (*n* = 5196) was divided randomly in two, a Kozak consensus logo was generated from one half, and Kozak scores were generated from the other half and for the bicistronic genes. Individual Kozak-like bit scores can be found in Supplementary Dataset S03 (S03 A-K)

**Start Codon 3-mer analysis**

To estimate the bias of “AUG” codon frequencies within a given sequence annotated as encoding a transcript, it was established that given a sequence of length *n*, there is a 0.25 probability for each nucleotide to be encountered at any individual position in a sequence with 50% GC content. Hence, the probability of encountering an “AUG” sequence by random chance is 0.015625 (0.25 *0.25 *0.25). The probabilities for G and C were further adjusted by dividing 0.25 by the respective GC content to generate adjusted probabilities (p) to account for sequence bias. For 5’ UTRs, this was calculated to be 54% for *C. reinhardtii* and 56% for *A. protothecoides.* For annotated CDS, this was calculated to be 69.9% for *C. reinhardtii* and 66.4% for *A. protothecoides.* Globally (for “before ORF 2” and “sum” predictions, these were determined to be 64.1% for *C. reinhardtii* and 63.8% for *A. protothecoides*. The FASTA files were then generated containing sequences for the following specified regions of bicistronic loci: 5’ UTR, ORF 1, inter-ORF sequence, and ORF 2. These were also generated for the ORFs of all monocistronic genes with an annotated 5’ UTR (*n* = 12,232 in *C. reinhardtii*, *n* =5180 in *A. protothecoides*) in addition to a separate FASTA containing only the sequences of the 5’ UTRs. Using an R script, individual sequence lengths and total “AUG” 3-mers were calculated for every sequence in the dataset. To account for a start codon where translation would canonically occur, a value of 1 was subtracted from any sequence annotated as an ORF. Calculated lengths and codon counts were further authenticated via manual curation of all bicistronic genes and 50 random genes from the monocistronic dataset. Using the formula [(*n* – 2) * *p*], the expected “AUG” frequency (*e*) was calculated for every sequence in the dataset. The actual observed value (*o*) obtained from the R script was then divided by the estimation to create a ratio representing the “AUG” bias (*o*/*e*) of a sequence based on its length. A detailed dataset containing the calculation template, sequence lengths, observed and estimated “AUG” counts, and the observed/expected values for all sequences used in this study can be found in Supplementary Dataset S04 (S04A-O).

**RNA-Seq and Ribo-Seq reanalysis**

RNA-Seq reads for *C. reinhardtii* from Strenkert et al. (main text reference 43) were downloaded from National Center for Biotechnology Short Read Archive (NCBI SRA) at accession number PRJNA445880. Ribo-Seq reads from Gotsmann et al. (main text reference 38) were downloaded from NCBI SRA at accession number PRJNA1019502. Each set of reads was mapped to the *C. reinhardtii* reference genome assembly (v6.1, available at https://phytozome-next.jgi.doe.gov/) with STAR (v2.4.0j) using --runThreadN 4 --alignIntronMax 5000. The resulting sam-formatted files were compressed, sorted and indexed with samtools (v1.16.1) using view -b -h, sort, and index, respectively. Ribo-Seq and RNA-Seq reads were assigned to ORFs with bedtools multicov (v2.30.0) using -s. The number of read counts per locus were normalized by ORF length in nt, and $\log_{10}$ transformed. The resulting values were plotted for ORF2 versus ORF1 for all high-confidence *C. reinhardtii* polycistronic loci with the ggplot2 package in R. The Pearson's correlation coefficient was calculated in R.

**Comparison of upstream Kozak scores with ORF 2 ribosomal occupancy.**

Ribo-Seq reads from *C. reinhardtii* were mapped to the reference assembly and assigned to loci as described above. Low coverage loci with fewer than 100 Ribo-Seq reads total (ORF 1 + ORF 2) were excluded from further analysis. Ribo-Seq read density was calculated by dividing the number of reads by the ORF length in nt. The ratio between the Ribo-Seq read density for ORF 2 relative to ORF 1 was calculated for each bicistronic locus. A single locus with an ORF 2 / ORF 1 ratio > 4 standard deviations from the mean was excluded from further analysis as an extreme outlier. Kozak sequence bit scores were calculated as described above for all AUGs upstream of ORF 2 in bicistronic loci, including those in the 5' UTR, ORF 1, and the inter-ORF sequence, regardless of reading frame. The Kozak sequence bit score for the highest scoring AUG was selected for each locus. The bicistronic loci were ordered by the maximum bit score and divided roughly into thirds as follows: highest (maximum Kozak score > 0.260, *n* = 7), medium (0.227 < maximum Kozak score < 0.260, *n* = 6), or lowest (maximum Kozak score < 0.227, *n* = 7). The distribution of ORF 2 / ORF 1 Ribo-Seq ratios were plotted as box plots for each of these groups

with the ggplot2 package in R. Statistical significance was calculated pair-wise for each comparison by Welch's t-test in R.

**Fluorescence and luminescence quantification and visualization**

Photoautotrophic and heterotrophic cultures of *A. protothecoides* were grown in 1 mL 24 well plates for a period of 128 hours before phenotyping, at which point $OD_{750}$ was measured for normalization with a Spectramax iD3 (Molecular Devices, LLC., San Jose, CA) well plate reader, which was also used for subsequent fluorescence and luminescence measurements. Before taking a measurement, cell plates were set to shake at a speed of "high" for 5 seconds. Venus fluorescence was measured using excitation-emission wavelengths of 515/555 nm. Superfolder GFP was measured using excitation-emission wavelengths of 488/528 nm. Luciferase activity measurements were conducted using the Pierce™ Gaussia Luciferase Flash Assay Kit. A working coelenterazine solution was made per manufacturer's instructions. For luminescence quantification, a 20 µL sample of cells were taken after well plate shaking and loaded into a black, clear bottom 96 well plate. A 50 µL working solution was then added to each biological replicate and relative luminescence was measured with the Spectramax iD3 at 487 nm. For visualization of luminescence, 200 µL of working solution was added to 1 mL culture of cells and gently mixed for a period of 10 minutes. Luminescence imaging was conducted using an Azure 200 Gel Imaging Workstation under the "chemiluminescence" setting with manual capture set to a period of 3 minutes. For both chemiluminescence

For confocal microscopy images, 50 µL aliquots were taken and centrifuged at a speed of 10,000 x g for 1 minute. Cells were then resuspended in 50 µL of 10 µM sodium phosphate buffer (pH = 7). 10 µL of each sample were then put on Fluorescent Antibody Slides (Thermo Scientific Cat. No. 3032) and covered with a Microscope Cover Glass (Fisherbrand 12-542A) and sealed with nail polish. Samples were observed under a Zeiss LSM880 Laser Scanning Confocal Microscope with a 63x oil objective lens (numerical aperture 1.15). Venus signal was observed with a laser excitation line at 514 nm (intensity, 20%); emission was collected between 519-599 (gain 650; offset 0). Superfolder GFP signal was observed with a laser excitation line at 488 nm (intensity, 20%); emission was collected between 510-550 (gain 700; offset 0). Chlorophyll fluorescence was observed with a laser excitation line at 635 nm (intensity, 4%); emission was collected between 647-721 (gain 650; offset 0). Corresponding brightfield images were acquired with a transmitted detection module with a Photon Counting PMT (T-PMT, gain 371; offset 0). Images were processed and color corrected using ImageJ software. The brightness and contrast range for chlorophyll was set between 10 and 150. The brightness and contrast range for Superfolder GFP was set between 0 and 100.

**PCR, cloning, plasmid assembly, cassette construction**

A detailed description of all primers, templates, vectors, plasmids, and assembly instructions used in this study can be found in Supplementary Dataset S06 (S06 A-G). In general, primers for fragment amplification were designed using the NEBuilder® Assembly Tool (https://nebuilder.neb.com/) and ordered from Integrated DNA Technologies (IDT, San Diego, CA). Codon optimization was performed according to a codon usage table from *Prototheca moriformis* UTEX 1435 (8) using *Gene Designer* (ATUM Bio Inc., Newark, NJ) software. Codon usage was based on the most frequently used codon, substituting less frequently used codons to avoid unwanted restriction sites, repeats and cryptic splice donor or acceptor sites. The *Venus* ORF was amplified from plasmid pLM005 (9) and then incorporated into other templates as described in S04. The codon-optimized *Gaussia princeps LUC* ORF was synthesized by GenScript Biotech, (Piscataway, NJ). All *in silico* cloning work was conducted using Geneious Prime by Dotmatics (Boston, MA).

PCR was performed using Herculase II Fusion DNA Polymerase (Agilent #600679). Thermocycler conditions were set according to the manufacturer's recommendations based on concentrations, target sequence length, and primer Tm (available in Supplemental Dataset 4 (S04)). PCR products were purified with an Omega Bio-Tek E.Z.N.A.® Gel Extraction Kit. Fragments and linearized vector backbones were assembled using the NEBuilder® HiFi DNA

Assembly mix and introduced into E. cloni® 10G Chemically Competent Cells using a 30 second heat shock method. Colonies were picked and grown overnight at 37°C with 250 rpm shaking in 2 mL of Luria Broth (LB) media with a concentration of 200 µg/mL ampicillin for selection. Plasmid purifications were conducted with an E.Z.N.A.® DNA Plasmid Mini Kit I (Omega Bio-Tek Inc. Norcross, GA), and concentrations were determined via NanoDrop (Thermo-Scientific NanoDrop™ One). Correct assembly and sequence were validated through Nanopore and Sanger sequencing (UC Berkeley DNA Sequencing Facility). For scale-up, 1-2 µL of verified plasmid minipreps were transformed into 10G competent cells and cultures were grown overnight at 37°C with 200-250 rpm shaking in 200 mL LB with 200 µg/mL ampicillin. Plasmid DNA maxipreps were performed using an E.Z.N.A.® DNA FastFilter Maxi Kit (Omega Bio-Tek Inc. Norcross, GA) per the manufacturer's instructions. DNA concentrations were measured with a NanoDrop One spectrophotometer (Thermo Fisher Scientific, Waltham, MA).

***A. protothecoides* strain, media, and culture conditions.**

*A. protothecoides* strain UTEX 250 was obtained from the UTEX Culture Collection of Algae (The University of Texas at Austin). UTEX 250-A was derived from a single colony isolated from UTEX 250. ApM1 culture media recipe was adapted from a composition described in US Patents US20140178950A1 (10) and US Patent US8927522B2 (11). Salts were added to 1 L MilliQ-purified water to achieve the following concentrations: 4.2 g (24.1 mM) potassium phosphate dibasic anhydrous (Fisher P288-500), 3.57 g (25.9 mM) sodium phosphate monobasic monohydrate (Fisher S369-1), 240 mg (974 µM) magnesium sulfate heptahydrate (Fisher M63-500), 250 mg (1.3 mM) citric acid (Fisher BP339-500), 1.7 mL (170 µM) of a 100 mM calcium chloride dihydrate stock solution (Fisher C79-500), 2 µM thiamine-HCl (Sigma T1270-25G), and 10 mL of 100X Ap trace element solution. A 1 L stock of 100X Ap trace element solution consisted of 2.743 g (14.3 mM) citric acid (Fisher BP339-500), 11 mg (14.3 µM) copper sulfate pentahydrate (Fisher BP346-500), 330 mg (340.5 µM) boric acid (Sigma B9645-500G), 1.4 g (4.89 mM) zinc sulfate heptahydrate (Sigma Z0251-500G), 948.5 mg (4.79 mM) manganese chloride tetrahydrate (Sigma M3634-100G), 3.9 mg (161 µM) sodium molybdate dihydrate (Sigma M1651-100G), and 110 mg (396 µM) iron sulfate heptahydrate (MP Biochemicals 194663). For solid media used in colony selection, 1.5% (w/v) agar (Fisher BP1423-500) was added to MilliQ water and then autoclaved before mixing with the other solutions to achieve correct concentrations. Cell cultures were started from a small loop or colony of the strain of interest and grown in either photoautotrophic or heterotrophic conditions. For photoautotrophic conditions, cultures were grown in ApM1 liquid media with the addition of 0.5% (w/v) glucose and 2 µM thiamine-HCl. Cultures were grown at 26°C and 50–100 µmol photons/$m^2$/s and shaken continuously at 140 rpm. For heterotrophic conditions, cultures were grown with ApM1 media with additional 2% (w/v) glucose. These cultures were also shaken continuously at 140 rpm but were grown in complete darkness. For transformant selection in either liquid culture or agar plates, G418 Sulfate (VWR E859-5G) was added at a concentration of 100 µg/mL.

***A. protothecoides* transformation and phenotyping**

Lithium acetate transformation was adapted from the protocol described in US Patent US-12037630-B2 (12). 1 M stock solutions of lithium acetate (Acros Organics, #6109-17-4), Tris-HCl (Fisher BP153-500), and polyethylene glycol 4,000 (PEG-4000, Alfa Aesar A16151) were made before starting the transformation. For cassette targeting, 100 µg of plasmid DNA diluted to a 750 µL solution was linearized by restriction digest with XbaI, (New England Biolabs) using recognition sites flanking the *DAO1* 5' and 3' targeting sequences. Digests were then extracted with 750 µL of 25:24:1 phenol-chloroform-isoamyl alcohol, pH = 8, (Fisher #BP17521-400) and centrifuged at maximum speed for 10 minutes in a tabletop centrifuge (Eppendorf). The aqueous phase was transferred to a new 1.5 mL Eppendorf microfuge tube, mixed with 525 µL isopropanol (Sigma-Aldrich W292907), and incubated at room temperature for 1 hour to precipitate the digested DNA. DNA was pelleted by centrifugation at maximum speed for 30 minutes; pellets were washed twice with 500 µL of 70% Ethanol (Sigma-Aldrich E7023) spinning for 5 minutes at maximum speed between each wash, and then dried in a sterile laminar flow hood. Linearized plasmid DNA was

dissolved in 50-100 µL of elution buffer (10 mM Tris-HCl, pH 7.5) (Omega Bio-Tek Inc. Norcross, GA). To prepare cells for transformation, a loop of UTEX 250-A wild-type cells from a plate culture was inoculated into 50 mL ApM1 media with 2% (w/v) glucose. Cultures were grown at 28°C in complete darkness with shaking at 140 rpm until an $OD_{750}$ between 1.75-3.5 was reached. Cultures were then transferred to 50 mL polypropylene conical tubes (Falcon 352098) and pelleted by centrifugation at 3750 rpm for 5 minutes in an Eppendorf 5810R centrifuge. Pelleted cells were washed with 5 mL of a 0.1 M lithium acetate/1X Tris-EDTA (TE) solution and centrifuged at 3750 rpm for 5 minutes. The cells were then resuspended in 500 µL 0.1M lithium acetate/1X Tris EDTA (TE) solution and shaken at 200 rpm for one hour in the dark. 150 µL cell suspension aliquots were incubated with 15 µg of linearized DNA for 30 minutes. Next, 750 µL of 0.1M lithium acetate/1X Tris EDTA (TE) / 40% PEG-4000 was added, and transformations were incubated overnight in the dark at a temperature of 27°C, shaking at 200 rpm. The next day, cells were collected via centrifugation and placed in ApM1 with 2% glucose and 1X thiamine media for an 8-hour recovery period. Afterward, cells were centrifuged to form a pellet, resuspended in 250 µL of 1M sorbitol, and plated onto ApM1 (with 2% (w/v) glucose, 2 µM thiamine-HCl, and 1.5% agar) selection plates with a concentration of 100 ng/µL G418. After a 7-to-14-day dark incubation period at 26°C, colonies were picked and grown heterotrophically in 1 mL ApM1 liquid cultures with 2% (w/v) glucose, 2 µM thiamine-HCl, and 100 µg/mL of G418 for selection. After 3-5 days, a 1/2500 serial dilution of the culture was conducted before replating on selective media for single colony isolation. Purified colonies that grew on these plates were isolated for further analysis.

For genotyping, DNA from wild type and transformant strains was extracted using a CTAB extraction method adapted from European Patent EP2785835A2. 2 mL of stationary phase wild type cultures were placed in a screw-capped tube, centrifuged at maximum speed, and then frozen at -20°C with a 4 mm glass bead (Fisher 11-312B). A mixture of 300 µL grinding buffer, 1.5 µL RNase A (VWR E866-1ML), and 250 mg of 0.5 mm glass beads (Fisher 35-535) was added, and the tube was shaken for 5 minutes in a bead beater. To this, 40 µL of 5M NaCl (VWR E529-500ML) and 66 µL 5% CTAB (VWR 0833-500G) were added, mixed by inversion, and incubated at 65°C for 1 hour. After incubation, samples were centrifuged at maximum speed for 10 minutes, and the supernatant was transferred to a fresh tube. To this, 300 µL of 25:24:1 phenol-chloroform-isoamyl alcohol pH 8 (Fisher BP17521-400) was added before centrifugation at maximum speed for 10 minutes. The aqueous phase was transferred to a new tube and mixed with 210 µL of isopropanol and incubated at room temperature for 1 hour. This mix was then centrifuged at max speed for 30 minutes to form a pellet. The pellet was then washed with 2 rounds of 500 µL 70% EtOH and dissolved in elution buffer (10 mM Tris-HCl. pH 7.5). DNA nanodrop concentrations were further diluted to have ideal genomic DNA concentrations around 100-300 ng/µL for use with the Herculase II Fusion DNA Polymerase (Agilent 600679). A detailed description of genotyping primers can be found in Supplementary Dataset 6 (S06).

**RNA extraction and RT-qPCR analyses**

For the collection of RNA, 50 mL photoautotrophic and heterotrophic cell cultures were harvested during mid-log phase of growth (~2.5 days). RNA extraction was carried out using the ZymoBIOMICS™ RNA Mini Kit (Zymo, R2001) per the manufacturer's protocol. To generate cDNA, 2.5 µg of extracted RNA were mixed with 1 µL of 10 mM dNTP mix (New England Biolabs), and 1 µL of 500 µg/mL oligo (dT)18 (New England Biolabs), then incubated at 65°C for 5 minutes. Reactions were cooled on ice, and a mix containing 4 µL first-strand buffer, 2 µL of 0.1 DTT (New England Biolab), 1 µL of RNasin (New England Biolabs), and 1 µL M-MLV (New England Biolab) was added to each sample. This was followed by a 50-minute incubation at 42°C and a 15-minute heat shock at 70°C. The resulting cDNA was then diluted 10-fold with MilliQ-grade water.

RT-qPCR primers specific to reporter ORF sequences were designed with the PrimerQuest™ Tool from Integrated Gene Technologies (IDT) to generate amplicon products less than 200 nt and close to or within the 3' end of the ORF. RT-qPCR reactions were conducted in 96-well plates using a CFX96 Real-Time System (BIO-RAD, Berkeley CA). A 20 µL reaction mix, consisting of 4 µL cDNA, 10 µL iTaq Universal SYBR® Green Supermix (BIO-RAD, Berkeley CA), and 2 µL each of reverse and forward primers, was added per well. 2-step amplification and melting curve cycles were

repeated 39 times with the denaturing step at 95°C and annealing/extension at 60°C. Primer efficiencies were resolved with a template sequence and five 10-fold dilutions. For absolute abundance, a plasmid (pMAD57) of known concentration, was used as a template for seven 10-fold dilutions. The molar concentration of the template was converted to $\log_2$ molecules and plotted against the Ct values to construct a standard dilution curve. The Ct values for each mRNA sample were then inputted into the line of best fit equation to calculate transcript abundance. All samples were run alongside a no-template control and a no-reverse transcriptase control. A detailed description of all RT-PCR and RT-qPCR primers can be found in Supplementary Dataset S05 (S05B &S05C).

**Analysis from Jacobebbinghaus et al. 2024**

In a previous study, the inter-ORF sequences taken from 22 bicistronic loci in *C. reinhardtii* were inserted between an *mVenus* reporter (ORF 1) and a promoter-less *aphVIII* drug-selectable gene (ORF 2) in a bicistronic expression vector (13). These expression vectors were then used to transform *C. reinhardtii*, placed under drug selection, and scored for *mVenus* expression to evaluate which of these candidate sequences (CSs) could facilitate the bicistronic co-expression of both *mVenus* and *aphVIII*. In this work, we reanalyzed that data to determine what contribution ORFs internal to the 22 candidate sequences (referred to in that work as CS1 - CS22) might have on co-expression. For each CS, a score was calculated as follows: first, the *mVenus* signal intensity from ORF 1 was summed from the percentage of transformants with low, medium, and high signal intensity as depicted in Jacobebbinghaus et al. Fig. 1b. CS22, which was reported as "n.a." was assigned a signal intensity of 0. Second, the transformation efficiency was determined from the length of each bar as depicted in Jacobebbinghaus et al. Fig. 1c. Finally, an efficiency score was calculated by multiplying these two values. The 22 candidate sequences (12, SI Data S1) were evaluated for the presence of additional ORFs. The 22 CSs were then sorted into six categories as follows: 1) those with no additional ORFs in the CS ($n$ = 6), 2) those with an ORF that begins and ends within the CS ($n$ = 2), 3) those with an ORF that is in-frame with ORF 2 ($n$ = 4), 4) those with an ORF that is 1 nt out of frame relative to ORF 2 ($n$ = 1), 5) those with an ORF that is 2 nt out of frame relative to ORF 2 ($n$ = 4), and 6) those with multiple and overlapping ORFs ($n$ = 5).

**Detection and functional annotation of proteins encoded by bicistrons**

Proteins from *C. reinhardtii* were identified by mass-spectrometry from a pool of experiments as described previously (1). Spectral counts that could not be assigned unambiguously to a single gene were filtered. The total count of the peptides was determined for each gene, and used to calculate the percentage of polycistronic ORF 1, polycistronic ORF 2, and monocistronic genes that had been detected. The same analysis was performed using only N-terminal or only C-terminal peptides.

The protein sequences of all ORFs in the bicistronic loci were exported as a FASTA file and analyzed for PFAM domains and Gene Ontology (GO) terms using InterPro 100.0 (https://www.ebi.ac.uk/interpro/). Subcellular localization predictions for all proteins were determined using DeepLoc 2.0 (14) (https://services.healthtech.dtu.dk/services/DeepLoc-2.0) Associated accessions, PFAM domain matches, InterPro and PANTHER GO terms, DeepLoc prediction scores, and any additional notes can be found in Supplementary Dataset S04.

**IRESFINDER analysis for *A. protothecoides***

To identify potential IRES-like activity in the bicistrons in *A. protothecoides* and *C. reinhardtii*, we used IRESfinder (15) to estimate IRES prediction scores for each species' bicistronic inter-ORF sequences. Bicistrons with either overlapping ORFs (no inter-ORF sequence) or an inter-ORF with less than 10 bp were filtered from the dataset. As a control, random stretches of intergenic sequence from *C. reinhardtii* and *A. protothecoides* were isolated with in-house scripts. Over 1000 sequences were selected at random from both species and matched for size to the polycistronic inter-ORF sequences. Additional controls for this analysis were selected from work by Weingarten-Gabbay et al. (16). In this work, the authors evaluated 55,000 short nt sequences from a range of sources in a high-throughput, bicistronic reporter assay to quantify the degree to which each

sequence can facilitate cap-independent translation. The full set of assayed sequences was filtered exactly as described in Wang and Gribskov (16) to exclude synthetic sequences and to include sequences with "splicing_score" > –2.5 and "promoter_activity" <0.2. From this reduced set, the 1000 sequences with the highest "IRES activity" were selected as the "IRES" set. For the "non IRES" set, 1000 sequences were randomly selected from the remaining 21,367 sequences that exhibited only baseline IRES function ("ires_activity" = 206.29). Each set of sequences described above was subjected to analysis by IRESfinder in mode 0 with default settings. The distribution of the resulting scores are plotted as a box plots with the ggplots2 package in R.

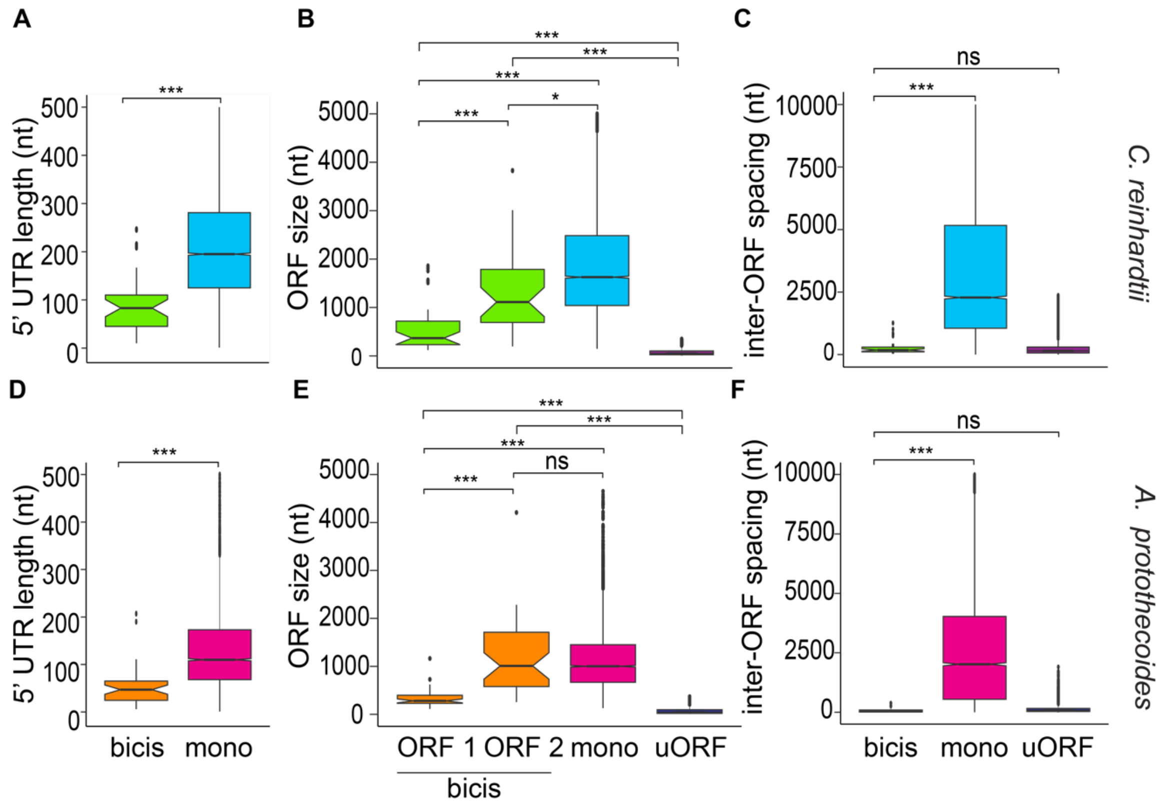


**Figure S1. Structural features of bicistronic loci.**

**(A and D)** The distribution of 5' UTR length in nucleotides. Highly conserved bicistronic genes (bicis) and protein coding monocistronic genes (mono). In *C. reinhardtii*, a sample size of *n* = 36 for bicistronic genes and *n* = 15,333 genes was used. In *A. protothecoides*, a sample size of *n* = 43 and *n* = 7698 was used. (**B and E)** Sizes of the protein coding ORFs in bicistronic genes (*n* = 36, 43), monocistronic genes (*n* = 15,333, 7,698), and uORFs (*n* = 31,774, 54,525) in *C. reinhardtii* and *A. protothecoides,* respectively. Bicistronic genes are separated based on their orientation as the upstream (ORF 1) or downstream (ORF 2) ORF relative to the 5' end of the transcript. (**C and F)** Distance between the stop and start codons of colinear gene pairs denoted as the "inter-ORF spacing" in *C. reinhardtii* and *A. protothecoides* respectively. The first plot denotes the length for all highly conserved bicistronic genes (*n* = 36, 43) genes. The second denotes colinear (adjacent genes on the same strand of the same chromosome with ≤ 20,000 nt between ORFs), monocistronic genes (mono, *n* =12,213, 7,698), and the third denotes uORFs within the 5' UTR of the transcript (uORFs, *n* = 29,210, 4,457). For all plots, whiskers indicate 1.5 times the interquartile range and notches indicate the confidence interval of the median. Outliers are plotted as individual points. Statistical significance was determined using Kruskal-Wallis and Wilcoxon rank-sum tests for sample comparison. Asterisks "*", "**", and "***" indicate *p*-values less than 0.05, 0.01, and 0.0001 respectively.

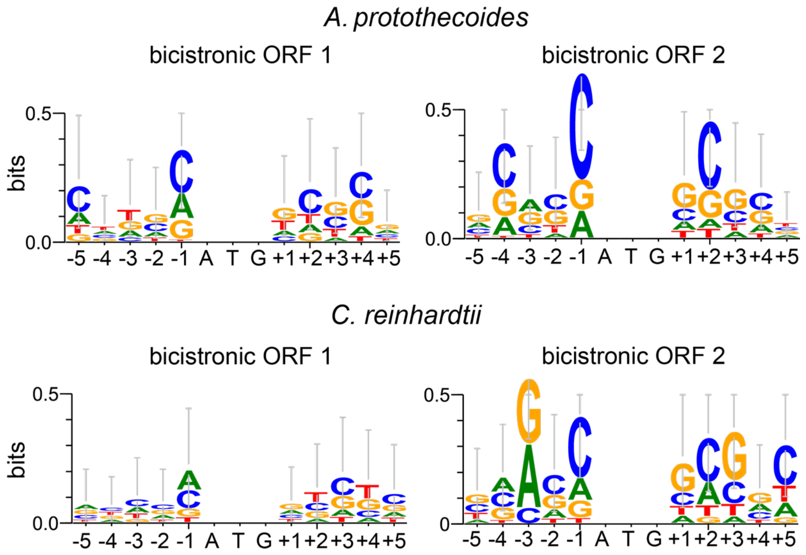


**Figure S2. WebLogos for the Kozak-like sequence of ORF 1 and ORF 2 in algal bicistrons**

WebLogos depicting the Kozak-like consensus sequence for ORF 1 and ORF 2 of all bicistrons in *A. protothecoides* (*n* = 43) and *C. reinhardtii* (*n* = 36).

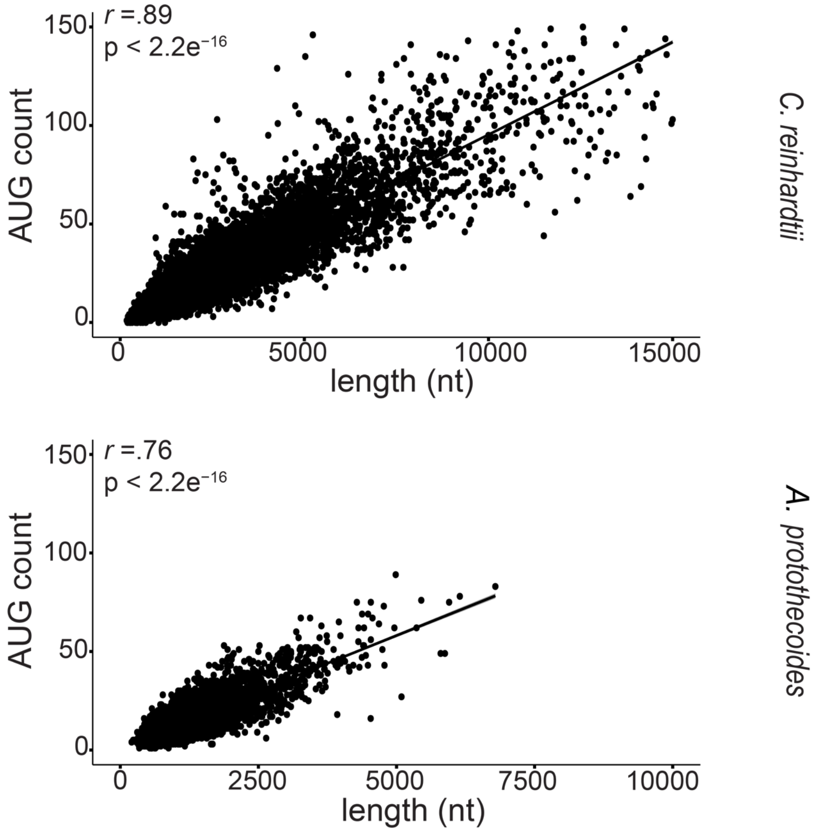


**Figure S3. Frequency of AUG codons correlates with increasing sequence length.**

The numbers of in and out of frame “AUG” sequences plotted against the sum of the lengths of the 5’ UTR plus the ORF sequences for a set of monocistronic genes in the genomes of *A. protothecoides* (*n* = 7,698) and *C. reinhardtii* (*n* =15,333). The line depicts a best fit linear regression. The correlation coefficient and *p*-value for significance are displayed at the top left of each graph.

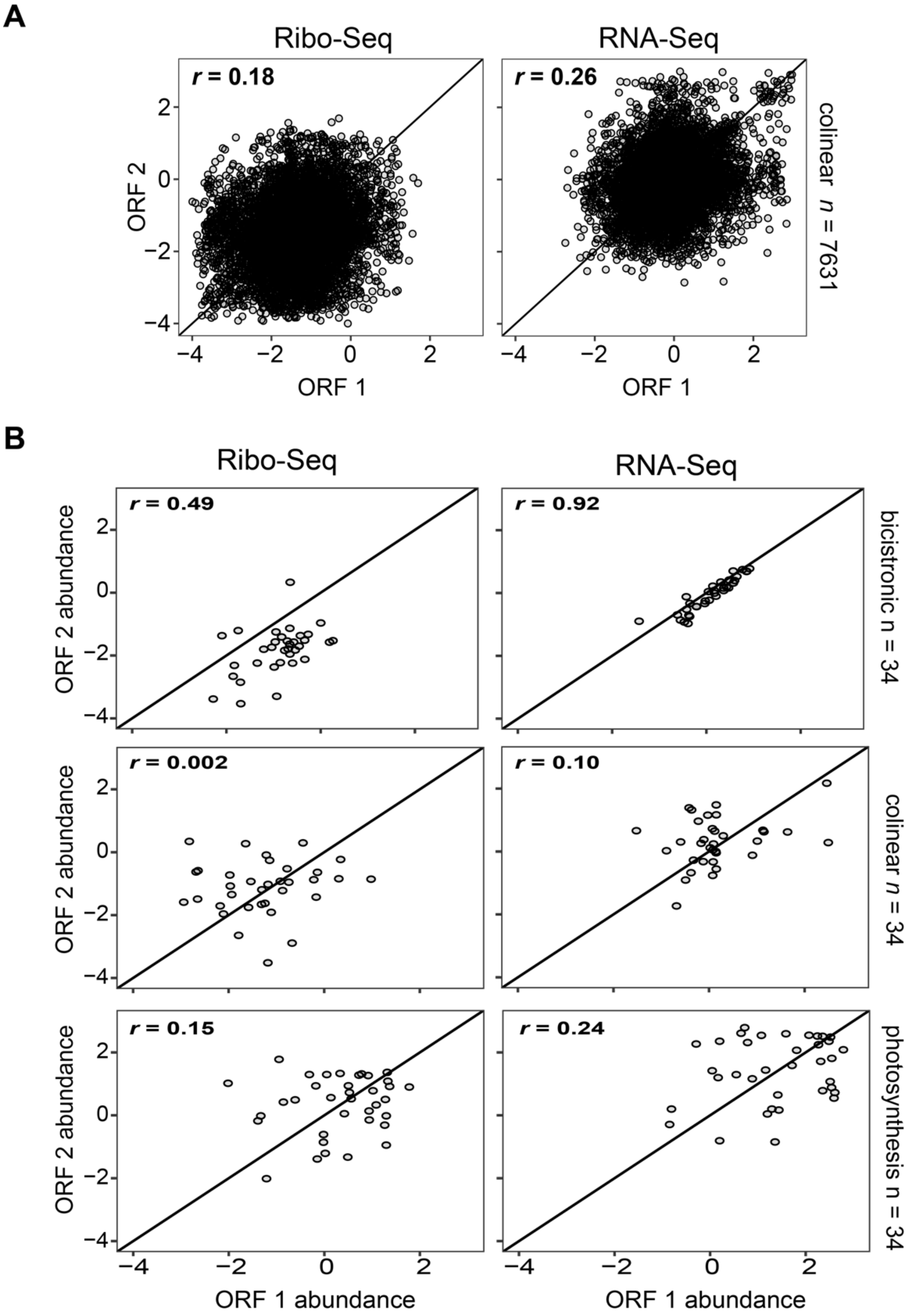


**Figure S4. Weak Correlation of RNA and abundance and ribosome occupancy in *C. reinhardtii***

**(A)** Correlation analysis of the population of colinear genes (*n* = 7,631) in *C. reinhardtii*, which are defined here as adjacent genes on the same strand of the same chromosome with ≤10,000 nt

between ORFs. A Pearsons correlation coefficient (*r*) is depicted in the upper left of each panel. A diagonal line representing a perfect 1:1 correspondence is plotted for reference. **(B)** Correlation analysis of RNA-Seq reads and Ribo-Seq reads for pairs of bicistrons (*n* = 34). $Log_{10}$ -normalized coverage scores (counts per nt of transcript) were plotted with ORF 1 (upstream) on the x-axis and ORF 2 (downstream) on the y-axis. A diagonal line representing a perfect 1:1 correspondence is plotted for reference. The same analysis was applied to monocistronic colinear genes which are defined here as adjacent genes on the same strand of the same chromosome with ≤10,000 nt between ORFs. Random sampling of 34 colinear gene pairs and their *r* values are presented here. Lastly, pairs of genes annotated by Gene Ontology to be in a common pathway (GO:0015979 photosynthesis) were paired randomly and analyzed as above (*n* = 36).

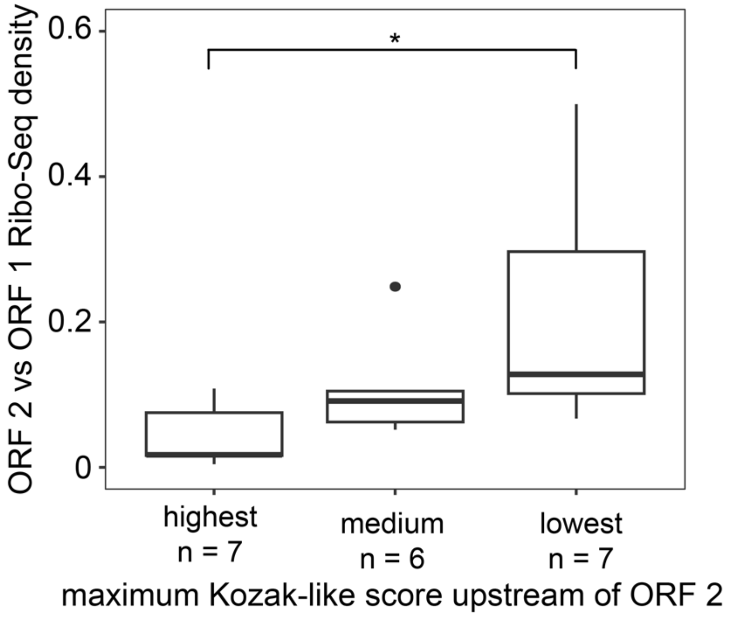


**Figure S5. Higher scoring Kozak-like sequences upstream of ORF 2 correlate with diminished ribosome occupancy in ORF 2 relative to ORF 1.**

To evaluate the contribution of Kozak-like sequences upstream of ORF 2 on translation, the bicistronic loci of *C. reinhardtii* were ranked into thirds based on the highest-scoring Kozak-like sequence upstream of ORF 2 as follows: highest (maximum score > 0.260), medium (0.227 < maximum score < 0.260), or lowest (maximum score < 0.227). For each locus, ribosomal occupancy was calculated as Ribo-Seq read density for ORF 1 and ORF 2 by normalizing the number of counts by the ORF length. The ratio of Ribo-Seq density between ORF 2 and ORF 1 was calculated for each locus, and the distribution of these ratios is plotted here for groupings of loci. Box plots depict the 25th, 50th and 75th percentiles. Whiskers indicate 1.5 times the interquartile range. Outliers are plotted as individual points. An asterisk indicates statistical significance ($p < 0.05$) as determined by Welch's t-test.

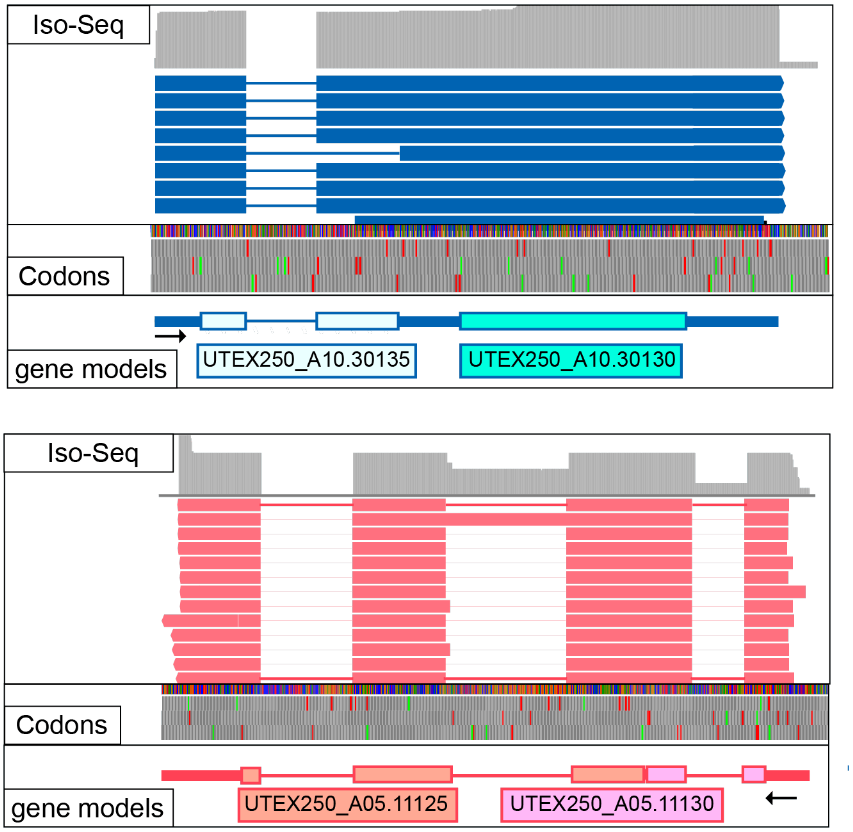


**Figure S6. Endogenous bicistronic loci for *in vivo* manipulation.**

IGV browser view of two endogenous bicistronic loci, predicted to encode *TOM22_SDHAF3* (UTEX250_A10.30135_UTEX250_A10.30130) and *OST4_FAM32A* (UTEX250_A05.11125_UTEX250_A05.11130). For Iso-Seq, the forward (+) strand is shown in blue, and the reverse (-) strand is in pink. The black arrow indicates the 5' to 3' orientation of the gene model. Coverage for each track is shown in grey. Stop and start codons are highlighted in red and green, all others are colored in grey. For gene models, a box indicates the exons of each ORF, a thick line indicates UTRs, and a thin line indicates introns. The regions upstream of the *SDHAF3* ORF contain no "AUG" sequences other than the start codon of *TOM22*. There is one "AUG" sequence upstream of the *FAM32A* ORF outside of the *OST4* start codon, located in the ORF of *OST4*.

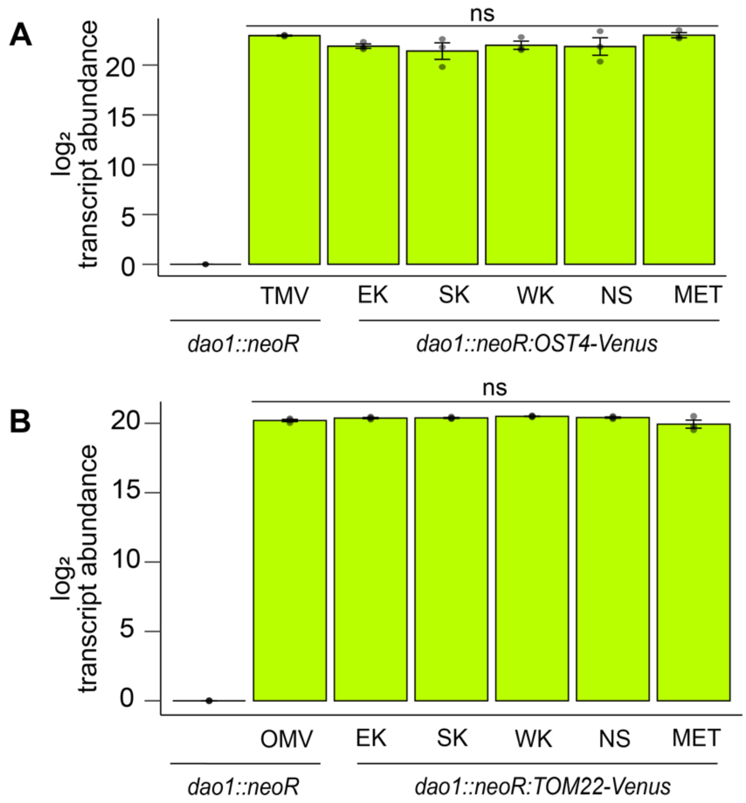


**Figure S7. Venus reporters show similar mRNA abundances**

Quantitative RT-qPCR analysis of **(A)** *OST4_Venus* and **(B)** *TOM22_Venus* reporter strains. Each bar represents the average of three biological replicates each averaged from technical duplicates. Error bars represent the standard deviation of the replicates ($n$ = 3), with “ns” indicating a $p$-value greater than 0.05 from a one-way ANOVA statistical test.

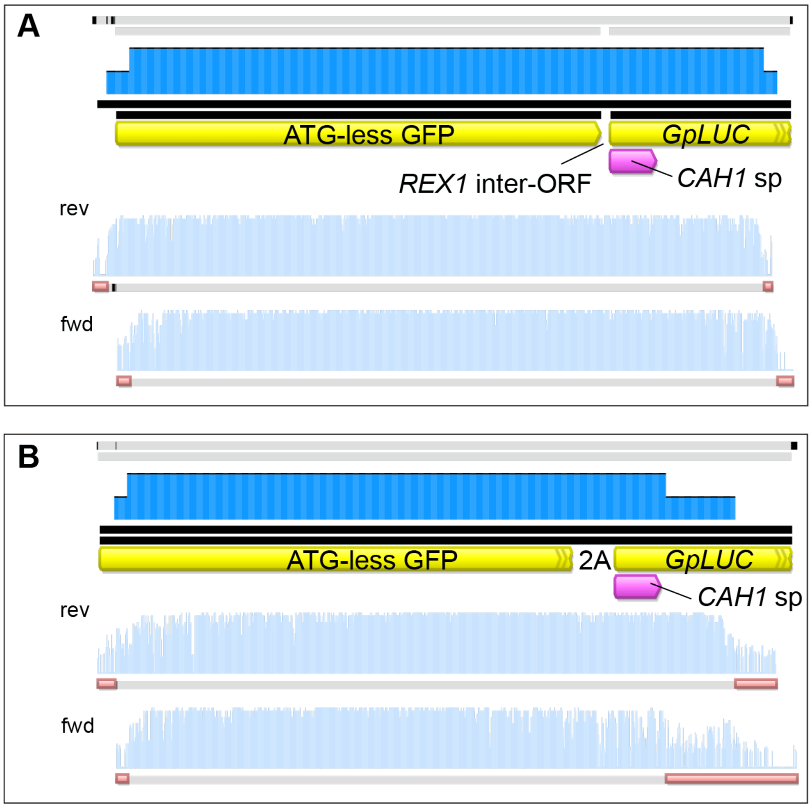


**Figure S8. Verification of synthetic bicistronic transcripts.**

Representative image of Sanger sequencing alignment of amplicons produced from RT-PCR of strains expressing bicistronic *GFP_LUC*. Yellow annotations denote the *GFP* and *Gaussia princeps LUC* sequences. The pink annotation encodes the signal peptide from *CAH1*, encoding a secreted carbonic anhydrase. In **(A)** the region between the coding sequences represents the 14 nt inter-ORF from *ApREX1S_ApREX1B*. In **(B)** the region between the coding sequences represents the 66 nt 2A peptide sequence.

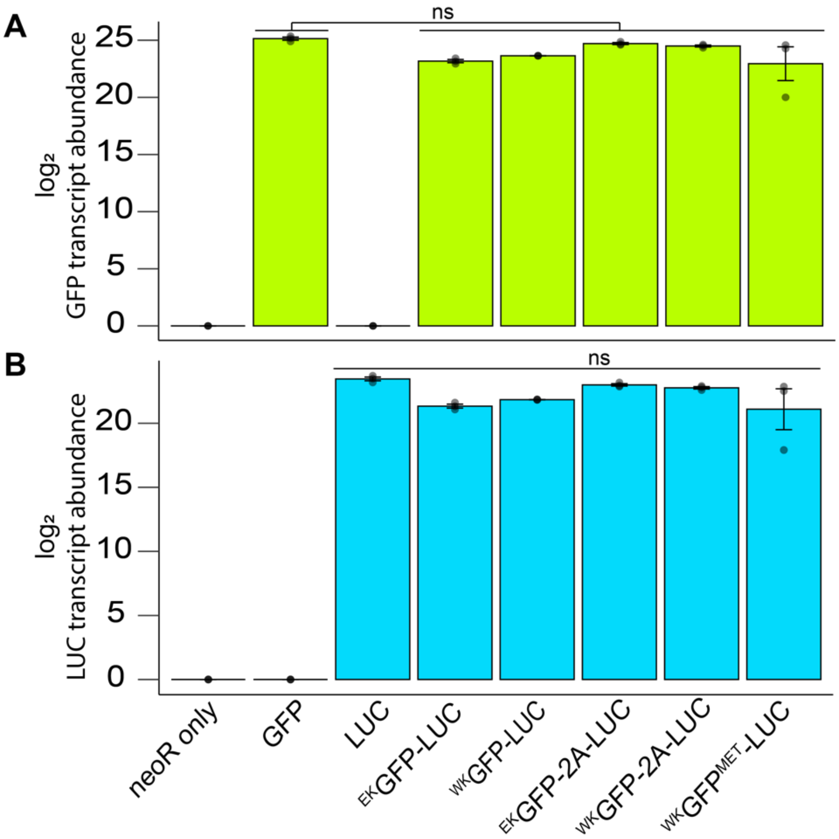


**Figure S9. Equivalent mRNA abundance of *GFP* and *LUC* reporters in bicistronic transcripts.**

Quantitative RT-qPCR analysis of the **(A)** *GFP* ORF and **(B)** *LUC* ORF of *GFP_LUC* dual reporter strains. Each bar represents the average of three biological replicates (average of technical duplicates). Error bars represent the standard deviation of the measurements ($n$ = 3), with "ns" indicating a $p$-value greater than 0.05 from a one-way ANOVA statistical test.

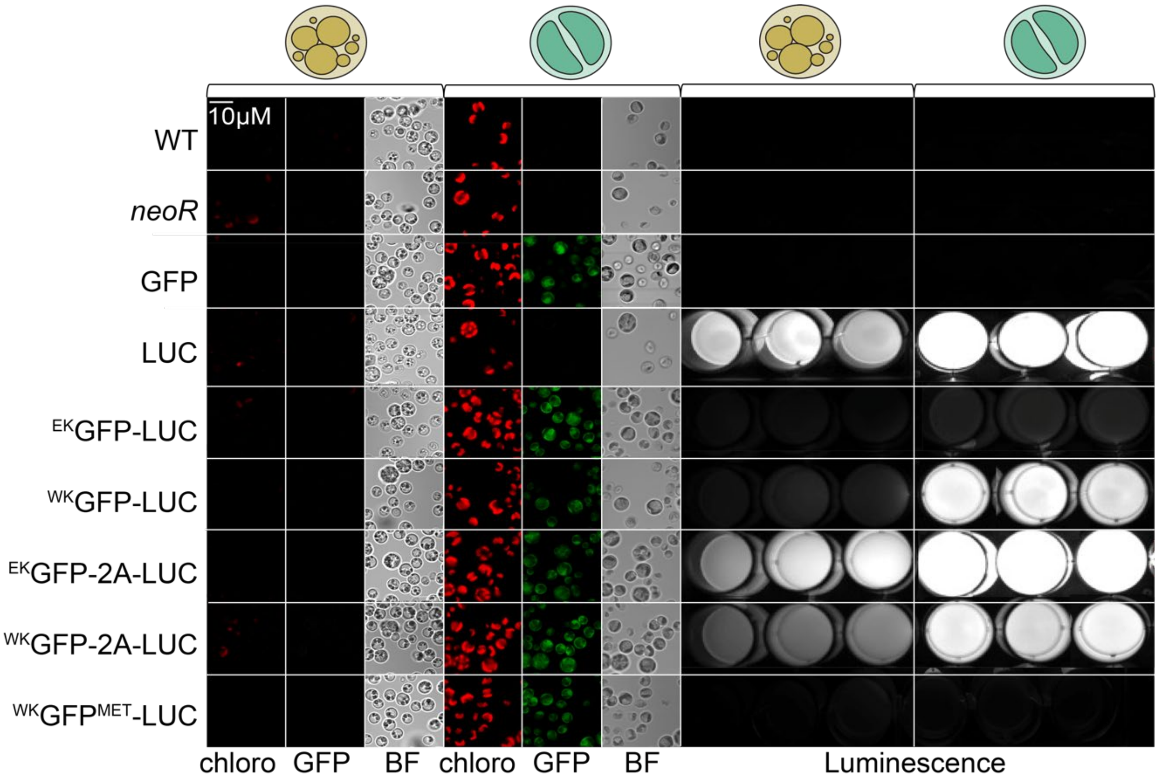


**Figure S10. Visualization of GFP and luciferase activity**

Representative confocal fluorescence microscopy and chemiluminescence images of dual reporter strains. For both heterotrophy (*pPSAD* ON) and photoautotrophy (*pPSAD* OFF), the left panels display chlorophyll fluorescence (633 nm excitation, 647-721 nm emission), GFP fluorescence (488 nm excitation, 510-550 nm emission), and the brightfield view, respectively. The scale bar (white, top left) applies to all microscope images. The right panels show luciferase chemiluminescence from three independent strains ($n$ = 3) for cells grown in heterotrophy and photoautotrophy. At the time of imaging, luminescence was normalized to the $^{\mathrm{WK}}$GFP-LUC strain.

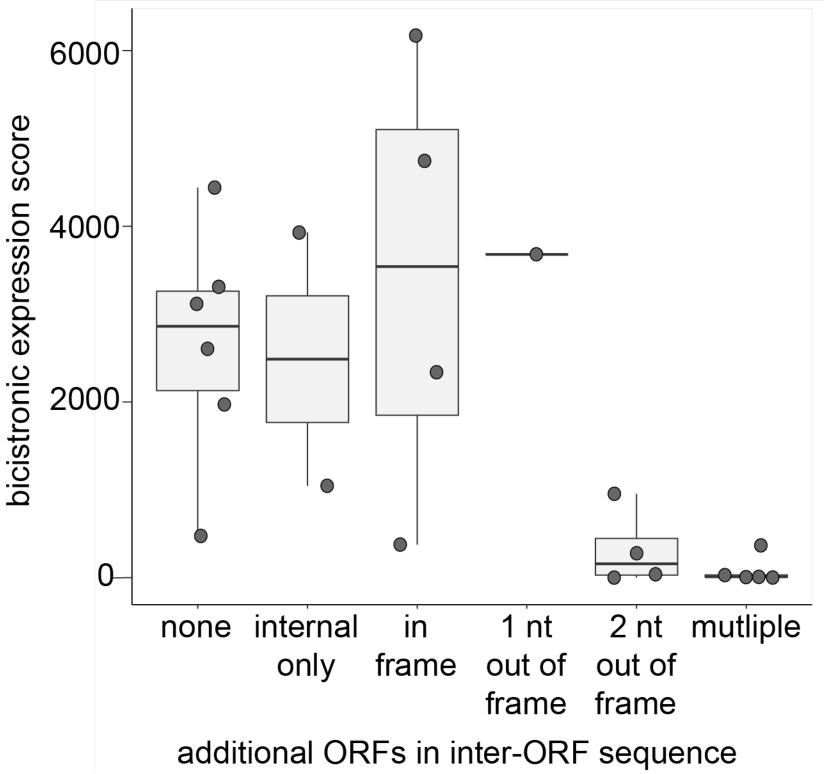


**Figure S11. The presence of additional ORFs in the inter-ORF region diminishes the bicistronic efficiency described in Jacobebbinghaus et al.**

In a prior study by from Jacobebbinghaus et al. (13), 22 constructs were compared for their ability to produce bicistronic expression after transformation into *C. reinhardtii*. All 22 constructs contained a *YFP* reporter gene in the ORF 1 position and an *aphVIII* drug-selectable marker in the ORF 2 position. The constructs differed only in the inter-ORF sequence, which they refer to as the candidate sequence (CS). Here, we re-evaluated the sequences of the 22 constructs from the prior study for the presence of additional ORFs within the CS, and grouped them as follows: 1) those with no additional ORFs in the CS, 2) those with an ORF that begins and ends within the CS, 3) those with an ORF that is in-frame with ORF 2, 4) those with an ORF that is 1 nt out of frame relative to ORF 2, 5) those with an ORF that is 2 nt out of frame relative to ORF2, and 6) those with multiple and overlapping ORFs. Each group is plotted relative to the bicistronic expression score calculated to reflect the combined expression of both ORF 1 and ORF 2 (see Methods).

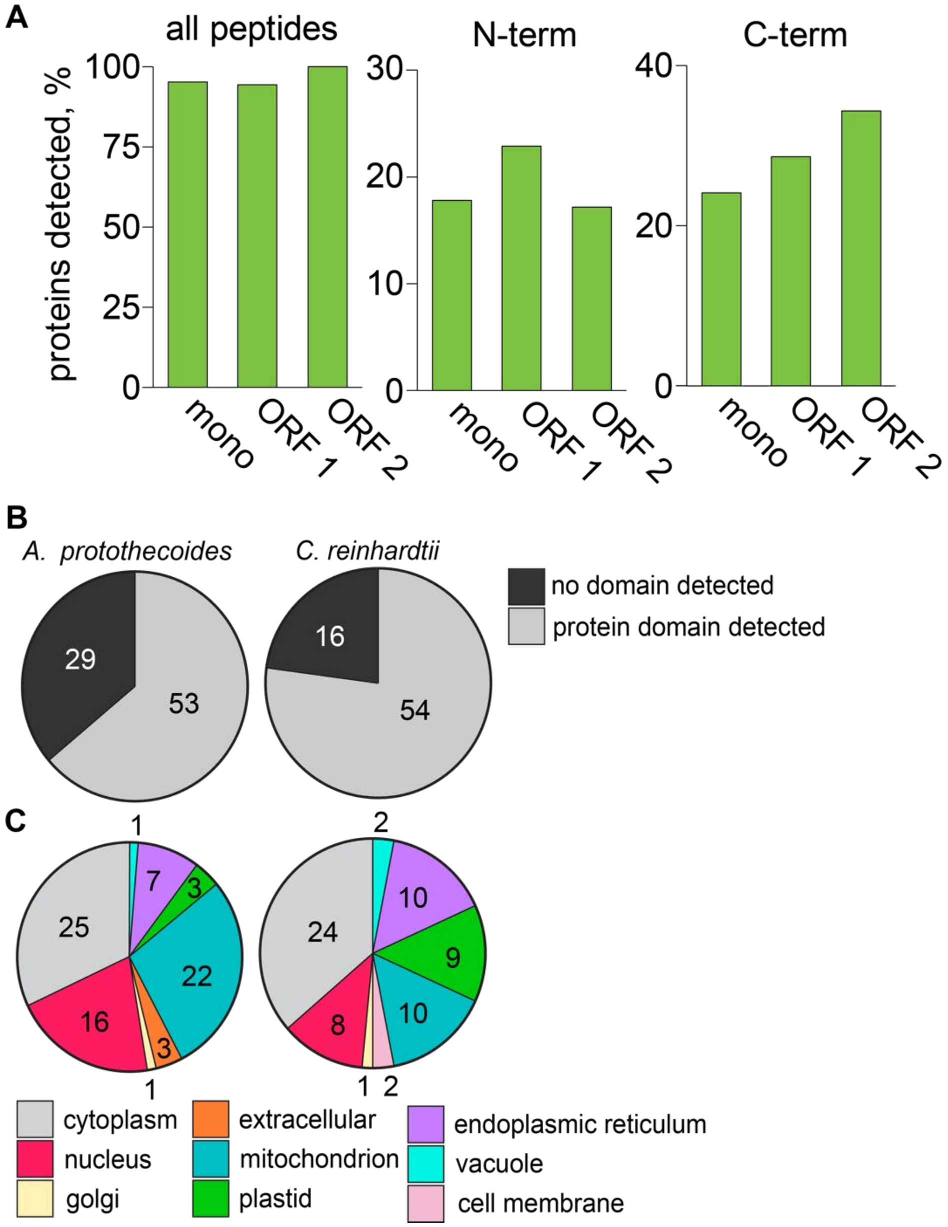


**Figure S12. PFAM and DeepLoc domain predictions of algal bicistronic loci.**

**(A)** Peptides from the proteome of *C. reinhardtii* were identified by mass spectrometry. The percentages of *C. reinhardtii* genes whose gene product was detected by at least one unambiguously assigned peptide for monocistronic (as "mono", *n* = 17,693), polycistronic ORF 1 (*n* = 35), and polycistronic ORF 2 (*n* = 35) genes are presented under “all peptides.” The percentage of proteins that were detected by an N-terminal or C-terminal peptide are broken out separately, as indicated. **(B)** Protein sequences from all bicistronic loci in this study were searched for conserved domains with InterPro scan. The number of proteins with an identified domain is presented as a pie chart for both *A. protothecoides* and *C. reinhardtii*. **(C)** Subcellular localization from all bicistronic loci in this study was predicted with DeepLoc 2.0 (13). The top prediction of each protein is presented for both *A. protothecoides* and *C. reinhardtii*.

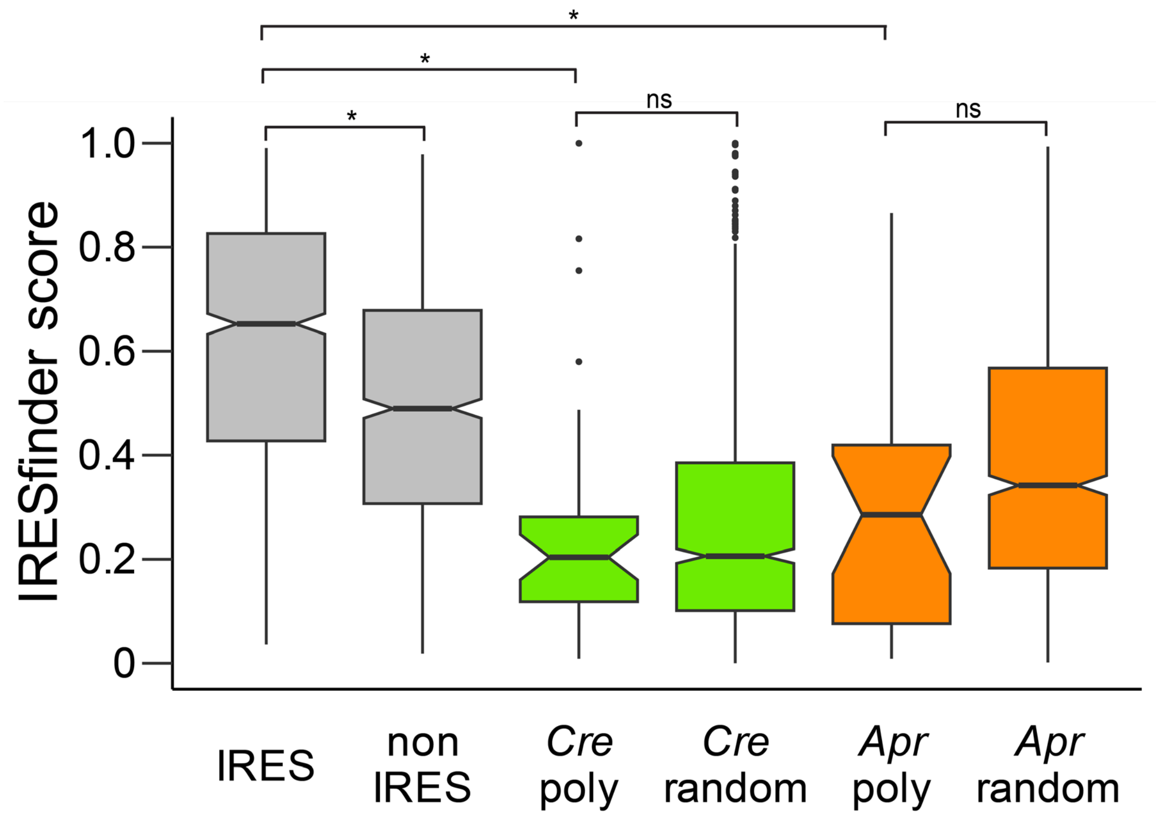


**Figure S13. Computational prediction of IRES function in *C. reinhardtii* and *A. protothecoides* bicistrons**

In order to systematically evaluate whether polycistronic inter-ORF sequences function as IRESs (i.e. facilitate cap-independent translation of ORF2), the inter-ORF sequences of high confidence *C. reinhardtii* and *A. protothecoides* polycistronic loci were subjected to analysis by a computational IRES prediction tool called IRESfinder. IRESfinder reports scores on a scale from 0 to 1 indicating increasing likelihood that the sequences will function as an IRES. The distribution of these scores is presented here for each set of sequences. As controls, sequences that had been empirically determined to function as IRESs ("IRES", $n$ = 1,000) or to not function as IRESs ("non IRES", $n$ = 1,000) were analyzed and their scores are plotted. All inter-ORF sequences >20 nt from the polycistronic loci of *C. reinhardtii* ($n$ = 35) and *A. protothecoides* ($n$ = 23) are plotted as "*Cre* poly" and "*Apr* poly", respectively. As an additional control, over 1,000 sized-matched sequences of random intergenic sequence from *C. reinhardtii* ($n$ = 1,050) and *A. protothecoides* ($n$ = 1,035) are plotted as "*Cre* random" and "*Apr* random", respectively. ANOVA was performed to determine which differences are statistically significant. All sets were significantly smaller than the IRES set ($p$ <0.01). *Cre* poly and *Apr* poly were not significantly different from their respective random counterparts. For each box plot, whiskers indicate 1.5 times the interquartile range, and notches indicate the confidence interval of the median. Outliers are presented as individual points.

## Supplemental Dataset Legends

**Supplementary Dataset S01.** Master list of bicistronic loci
Detailed information on bicistronic loci identified in *C.reinhardtii* and *A. protothecoides* including gene ID, coordinates, ORF size, inter-ORF spacing, and start codon counts.

**Supplementary Dataset S02.** Conservation chart for bicistronic loci
Data for all conserved bicistronic loci as described in figure 1. For each pair of hits in each species with a high scoring pair, the protein ID (or "unannotated" for putative proteins encoded by unannotated ORFs), the coordinates of the gene encoding that protein in the corresponding genome assembly, the BIT score of the match, supporting evidence (either colinear ORFs, EST support, or Iso-Seq support) and the search algorithm used are included.

**Supplementary Dataset S03.** Kozak-like scores and sequences
Kozak-like bit scores and sequences for all annotated and potential alternative start sites within the bicistrons and monocistrons of both *A. protothecoides* and *C. reinhardtii*.

**Supplementary Dataset S04.** AUG 3-mer analysis
The template used to calculate the AUG bias frequency in the bicistronic and monocistronic transcripts in *C. reinhardtii* and *A. protothecoides*. The values for all components used in this study can be found in the following sheets. The first column details the Gene ID. The second denotes base sequence length. The third denotes AUG 3-mer prediction based on 50% GC bias. The fourth is adjusted for GC bias of each species. The fifth column denotes the observed number of AUG 3-mers found in the sequence.

**Supplementary Dataset S05.** Bicistronic-encoded protein predictions
PFAM protein prediction, GO Ontology, and subcellular localization prediction for all proteins encoded on bicistronic genes in *A. protothecoides* and *C. reinhardtii*.

**Supplementary Dataset S06**. Master List of primers, plasmids, templates and amplicons

All primers and plasmids used in assembly, genotyping, and RT-qPCR analysis within this study, as well as expected amplicon sizes for genotyping and plasmid assembly.

## Supplementary References


1. S. D. Gallaher, et al., Widespread polycistronic gene expression in green algae. Proc. Natl. Acad. Sci. U.S.A. **118**, e2017714118 (2021).
2. Craig, Rory et al. (Forthcoming 2024). UTEX250 Genome and Iso-Seq reads for Leaky ribosomal scanning enables tunable translation of bicistronic ORFs in green algae. [Dataset]. Dryad. https://doi.org/10.5061/dryad.j9kd51cn3
3. H. Thorvaldsdottir, J. T. Robinson, J. P. Mesirov, Integrative Genomics Viewer (IGV): high-performance genomics data visualization and exploration. Briefings in Bioinformatics **14**, 178–192 (2013).
4. S. Suzuki, R. Endoh, R. Manabe, M. Ohkuma, Y. Hirakawa, Multiple losses of photosynthesis and convergent reductive genome evolution in the colourless green algae Prototheca. *Sci Rep* **8**, 940 (2018).
5. D. M. Emms, S. Kelly, OrthoFinder: solving fundamental biases in whole genome comparisons dramatically improves orthogroup inference accuracy. Genome Biol **16**, 157 (2015).
6. F. R. Cross, Tying Down Loose Ends in the *Chlamydomonas* Genome: Functional Significance of Abundant Upstream Open Reading Frames. *G3 Genes|Genomes|Genetics* **6**, 435–446 (2016).

7. G. E. Crooks, G. Hon, J.-M. Chandonia, S. E. Brenner, WebLogo: A Sequence Logo Generator: Figure 1. *Genome Res.* **14**, 1188–1190 (2004).
8. L. C. M. Mackinder, *et al.*, A repeat protein links Rubisco to form the eukaryotic carbon-concentrating organelle. *Proc. Natl. Acad. Sci. U.S.A.* **113**, 5958–5963 (2016).
9. Franklin, S. et al. Recombinant microalgae cells producing novel oils (2011). US Patent US7935515B2, filed November 30th, 2009, granted May 3rd, 2011
10. Coragliotti, A. et al. Microalgal polysaccharide compositions (2015). US Patent US8927522B2, filed March 29th, 2010, granted January 6th, 2015.
11. Franklin, S. et al. Tailored oils produced from recombinant heterotrophic microorganisms. (2014). US Patent US8765424B2, filed July 6, 2012, granted July 1, 2014.
12. Moseley et al. Production of lipids and terpenoids in Auxenochlorella protothecoides (2021). US Patent US12037630B2, filed November 5th, 2021, granted July 16th, 2024.
13. N. Jacobebbinghaus, K. J. Lauersen, O. Kruse, T. Baier, Bicistronic expression of nuclear transgenes in *Chlamydomonas reinhardtii*. *The Plant Journal* tpj.16677 (2024). https://doi.org/10.1111/tpj.16677.
14. Thumuluri, V et al. DeepLoc 2.0: multi-label subcellular localization prediction using protein language models. *Nucleic Acids Research*, Web server issue 2022.
15. Zhao et al. IRESfinder: Identifying RNA internal ribosome entry site in eukaryotic cell using framed k-mer features *Journal of Genetics and Genomics* **45**, 403-406 (2018).
16. S. Weingarten-Gabbay, *et al.*, Comparative genetics: Systematic discovery of cap-independent translation sequences in human and viral genomes. *Science (80-. ).* **351** (2016).
